# Sensor Node Deployment Optimization for Continuous Coverage in WSNs

**DOI:** 10.3390/s25123620

**Published:** 2025-06-09

**Authors:** Haris Muhammad, Haewoon Nam

**Affiliations:** Department of Electrical Engineering, Hanyang University, Ansan 15588, Republic of Korea

**Keywords:** particle swarm optimization (PSO), adaptive search factor, fast convergence, wireless sensor network, Delaunay triangulation

## Abstract

Optimizing sensor node coverage remains a central challenge in wireless sensor networks (WSNs), where premature convergence and suboptimal solutions in traditional optimization methods often lead to coverage gaps and uneven node distribution. To address these issues, this paper presents a novel velocity-scaled adaptive search factor particle swarm optimization (VASF-PSO) algorithm that integrates dynamic mechanisms to enhance population diversity, guide the search process more effectively, and reduce uncovered areas. The proposed algorithm is evaluated through extensive simulations across multiple WSN deployment scenarios with varying node densities, sensing ranges, and monitoring area sizes. Comparative results demonstrate that the approach consistently outperforms several widely used metaheuristic algorithms, achieving faster convergence, better global exploration, and significantly improved coverage performance. On average, the proposed method yields up to 14.71% higher coverage rates than baseline techniques. These findings underscore the algorithm’s robustness and suitability for efficient and scalable WSN deployments.

## 1. Introduction

Wireless sensor networks (WSNs), empowered by compact micro-electromechanical system (MEMS) technologies, are widely used in applications such as environmental monitoring and disaster management. These networks face persistent challenges, including energy constraints and coverage optimization, especially in dynamic and inaccessible environments [1]. Unlike traditional networks, WSNs operate autonomously without fixed infrastructure and are specifically designed to monitor and communicate various physical or environmental parameters, including temperature, motion, noise, pressure, vibration, and pollution [2]. These networks rely on a central entity commonly referred to as a sink or base station that acts as a bridge between users and the network, enabling data access and query functionality. A typical WSN comprises hundreds or even thousands of sensor nodes, requiring advanced and adaptive protocol designs to effectively operate within dynamic environments and limited resource conditions [3]. Over the past decade, there has been a significant surge in research, standardization efforts, and industrial investment in WSN technologies [4]. WSNs are now integral to a wide range of applications, particularly in scenarios where human access is limited, such as environmental monitoring, disaster recovery, pollution detection, military surveillance, healthcare systems, and smart home environments [5].

Each sensor node in a WSN is capable of independently collecting, storing, processing, and exchanging environmental data with neighboring nodes [6]. However, coverage gaps in areas not monitored by any sensor can emerge when regions fall outside the sensing range. To address this, researchers have proposed integrating mobile nodes with energy-efficient metaheuristics like the scalable firefly algorithm, which enhances coverage by dividing the network into manageable cells and enabling accurate estimation of physical coverage [7]. Additionally, hybrid optimization frameworks, such as those combining branch-and-cut algorithms with reinforcement learning, have been developed to ensure comprehensive coverage. To further improve network performance and mitigate interference, effective strategies for beamforming and re-optimization are crucial [8]. A notable contribution in this area is the wireless sensor network hole detection (WHD) technique, which quantifies the presence of coverage holes in randomly deployed sensor fields within regions of interest (ROIs) [9]. This method incorporates metrics such as average hole detection time to balance energy efficiency with quality of service (QoS) objectives [10].

Current research primarily focuses on developing computationally and energy-efficient algorithms and protocols, particularly for monitoring and control applications [11]. Despite their capabilities, sensor nodes are constrained by limited battery life, processing power, and memory for data transmission and detection [12]. The accuracy and comprehensiveness of the data collected improve when sensors are positioned closer to the observed events. Since their introduction, WSNs have found extensive use in security and surveillance, as well as in industries like factory automation, military operations, remote patient monitoring, environmental observation, and disaster prediction [13,14,15,16,17]. Sensor nodes play a critical role in these applications, gathering essential environmental data and transmitting it to the base station (BS) or sink via multicast communication [18]. However, the network’s structure may increase energy consumption in nearby nodes, potentially causing energy depletion or “holes” within the network [19]. Generally, WSNs consist of numerous sensor nodes transmitting environmental data to a base station for aggregation and analysis.

Coverage maintenance remains a key challenge due to battery limitations and node failures. Several lightweight distributed methods have been proposed to detect and address coverage gaps using mobile nodes, though many incur significant communication overhead [20,21]. In WSNs, a two-step process is typically employed to maintain coverage. First, a lightweight distributed system enables nodes to assess their environment and identify coverage gaps [22]. Once the number of gaps exceeds a certain threshold, the algorithm selects and prioritizes mobile nodes to fill these gaps, focusing on those closest to the gaps and with the highest priority. Over the past decade, advancements in embedded micro-sensing and wireless communication technologies have led to the development of increasingly compact and affordable wireless devices [23]. Several techniques leverage nodal attributes related to network topology and geometric connectivity to detect coverage gaps. However, most algorithms require a high average node degree to identify specific patterns, resulting in significant communication overhead [24]. Notably, while these methods can identify nodes near coverage gaps, they do not provide efficient clustering mechanisms, which could simplify coverage maintenance. This high communication cost, unfortunately, shortens the lifespan of WSNs considerably. Similarly, our PSO-based approach enhances sensor placement and coverage in WSNs to improve overall network performance. Recent research on active reconfigurable intelligent surface (RIS)-aided non-orthogonal multiple access (NOMA)-enabled space–air–ground integrated network (SAGIN) with UAVs highlights advanced energy-efficient optimization strategies [25]. Similarly, our PSO-based approach enhances sensor placement and coverage in WSNs to improve overall network performance.

Extending network lifetime is challenging due to limited data speed and the difficulty of replacing or recharging batteries in randomly dispersed sensor nodes (SNs), often in inaccessible areas [26]. Moreover, energy consumption increases more than linearly with communication distance, underscoring the critical need for efficient energy management strategies. In multi-hop communication systems, sensor nodes located near the sink experience disproportionately heavy traffic loads, causing their energy to deplete faster—this phenomenon is commonly referred to as the “Energy Sink-Holes Problem” or “Bottleneck Problem” [27,28]. To address coverage optimization, a perception model combining path loss and false alarm probability has been developed [29]. Furthermore, a novel multi-objective optimization framework has been proposed for heterogeneous-function WSNs, balancing coverage, connectivity, and cost. This approach introduces an additional cost dimension to accommodate the distinct monitoring requirements of various functional sensors within specific areas. By employing an efficient node deployment strategy, redundancy and costs are reduced while simultaneously improving network coverage, robustness, energy efficiency, and load balancing, ultimately contributing to an extended network lifetime [30]. To maximize coverage, minimize the number of deployed nodes, and maintain network connectivity, the proposed method is based on a novel optimization approach called the Nutcracker Optimization Algorithm (NOA) [31]. Reliable Clustering with Optimized Scheduling and Routing for Wireless Sensor Networks introduces a novel GridCosins Chain Clustering technique that uses GridCosins distance for node grouping and forms distance-based tree topologies, reducing transmission range and enhancing network lifetime [32].

Ensuring complete coverage in WSNs is a complex task that has attracted considerable research focused on developing effective solutions to close coverage gaps, improve network efficiency, and enhance overall performance. As a result, there have been many recent research developments. Multi-objective coverage algorithms for WSNs that optimize energy-saving and coverage rate metrics are noteworthy advances [33]. The whale optimization algorithm (WOA), which draws inspiration from the hunting strategies of whales, has also been used to optimize WSNs [34]. Reverse learning has been included to increase network coverage. Furthermore, it has been suggested that Lévy flights be included in WOA to guarantee extensive network coverage. The bee algorithm, particle swarm optimization algorithm, weed algorithm, wolf pack algorithm, glowworm swarm optimization, simulated annealing (SA), ant colony optimization (ACO), improved grey wolf optimizer with multi-strategies, improved cuckoo search, and social spider optimization (SSO) are some of the other intelligent optimization algorithms that have been investigated. Together, these various algorithms help to advance coverage tactics in WSNs.

Recent research has demonstrated the effectiveness of PSO-based techniques in WSN optimization, particularly in enhancing localization accuracy through error minimization and sensor placement optimization [35]. In reconfigurable WSNs (RWSNs), PSO-based algorithms address complex multi-peak optimization problems, improving sensor deployment efficiency and coverage quality [36]. Self-adaptive estimation PSO (SEPSO) further refines sensor placement by reducing computation time compared to traditional methods like the virtual force approach [37]. In heterogeneous WSNs (HWSNs), PSO-based methods enhance energy efficiency and convergence speed while minimizing redundancy and coverage gaps [38]. Deploying sensors in 3D WSNs presents additional challenges due to higher deployment costs and connectivity constraints, where PSO variants such as virtual force-directed PSO (VFPSO) have been proposed to maximize coverage while minimizing energy consumption [39]. Moreover, integrating PSO with variable domain chaos optimization (VDCOA) has been shown to improve network efficiency, sensor distribution, and coverage rates [40]. In WSN clustering, PSO has been utilized for optimal cluster head selection by leveraging hybrid PSO and K-means clustering to enhance network lifespan and balance energy consumption [41].

Particle swarm optimization, developed over the past two decades, offers significant advantages over previous intelligent optimization techniques, including simpler iteration rules and faster convergence rates [42]. This optimization algorithm has been effectively implemented across various engineering domains. For instance, co-evolutionary PSO algorithms have been employed to explore dynamic deployment optimization in WSNs [43]. Optimization algorithms play a crucial role in enhancing WSN’s performance. The hybridization of the global PSO algorithm with shuffled frog leaping optimization has resulted in a more effective problem-solving approach [44]. Furthermore, hybrid algorithms integrating PSO with Genetic Algorithms have been developed to maximize WSN coverage. At the same time, a combination of PSO and simulated annealing has been proposed for energy-efficient coverage optimization [45].

### Motivation

**Figure 1:**
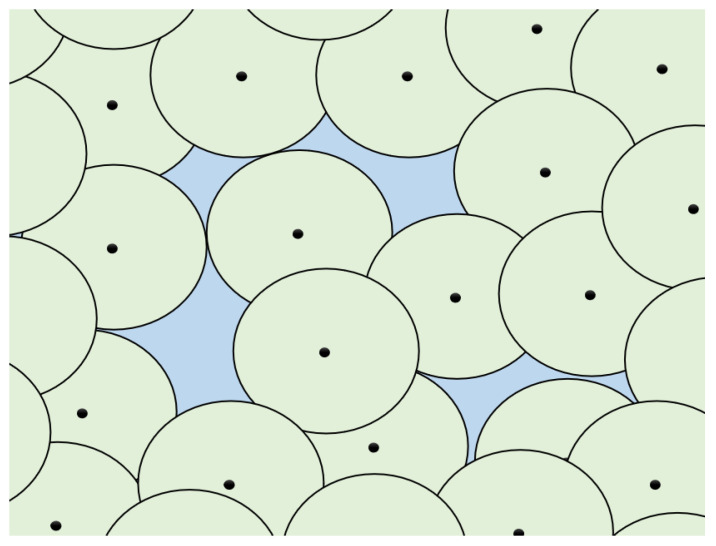
Coverage hole problem in WSNs.

WSNs are widely used in applications that require reliable and high-quality coverage, such as environmental monitoring, surveillance, and industrial automation. However, achieving uniform sensor distribution and complete area coverage remains a significant challenge. Factors like irregular node placement, limited energy resources, and inefficient deployment strategies often result in coverage gaps and uneven sensing performance. Many existing optimization methods, particularly those based on PSO, struggle with issues such as premature convergence and getting trapped in local optima. These limitations can lead to poor sensor placements and large uncovered areas, which in turn disrupt both sensing and communication, as illustrated by the light blue-highlighted region in Figure 1.

To address these challenges, this paper proposes a velocity-scaled adaptive search factor PSO algorithm, which improves the global search ability and speeds up convergence. The algorithm begins with Hammersley sequence-based initialization to ensure an even initial distribution of nodes, and it uses Delaunay triangulation to effectively detect and resolve coverage holes. The main goal of this research is to offer a robust and efficient optimization technique that ensures maximum coverage, uniform sensor placement, and improved search accuracy. By overcoming the key drawbacks of earlier approaches, the proposed VASF-PSO significantly enhances WSN coverage performance and offers a practical solution for real-world deployments.

## 2. Related Work

A comprehensive approach integrates deployment, detection, and network models to address coverage gaps with varying characteristics. The framework aims to enhance coverage quality, extend network lifespan, and reduce sensor usage under uncertain parameters [46]. It employs planning, selection, movement, and decision-making techniques, categorized into distribution, centralization, stochastic, heuristic, approximation, and classical algorithms [47]. An improved particle swarm optimization algorithm (NS-IPSO) is also introduced to efficiently segment sensor nodes by enhancing distance estimation accuracy between unknown and anchor nodes, enabling better regional division. This approach identifies sensor nodes with shorter paths to anchor nodes, ensuring optimal placement. A surveillance network monitors partial discharges in high-voltage equipment using ultrasonic sensors designed for 3D alignment. A secure sensor cooperation strategy enhances target acquisition, reduces delays, and saves energy [48]. Advanced algorithms, including Gaussian and polynomial kernel integration and graph-based manifold learning, enable precise localization by mapping physical spaces to high-dimensional data. Their performance has been validated under various conditions. A distributed self-healing mechanism also detects and closes coverage gaps, accurately predicting their size and location. Furthermore, by predicting the amount and location of coverage gaps, a distributed self-healing system automatically finds and fixes them, improving the resilience of networks without the need for centralized control.

WSNs optimize coverage and minimize energy use by coordinating node movements to fill gaps without disrupting connectivity. Sensor nodes share maintenance data with the cluster head (CH), selected based on cooperation rank, transmission rank, and residual energy [49]. Mobile nodes gather detection data via intermediary channels, reducing latency and energy consumption. A flexible, energy-efficient protocol addresses QoS challenges by accommodating sensor resource constraints. To estimate coverage gaps, a computational geometry method uses Delaunay triangulation and empty circle attributes to identify and assess gaps in large-scale networks [50].

### Contributions

This paper proposes the velocity-scaled adaptive search factor PSO algorithm, which dynamically adjusts the search factor based on the distance of a particle from the swarm’s optimal position, effectively balancing exploration and exploitation. In the PSO algorithm, the search factor controls the impact of the particle’s previous velocity on its current velocity, thereby balancing local and global exploration of the search space. Local exploration focuses on finding the best solutions near the particle’s current position, while global exploration seeks optimal solutions across the entire search space. A lower search factor facilitates local exploitation, while a higher search factor promotes global exploration.

The contributions of the paper are summarized as follows:

1. Adaptive Algorithm: This paper proposes a new PSO variant where the search factor is dynamically scaled based on particle velocity relative to the global best. Unlike conventional PSO or previous adaptive methods, our algorithm increases the exploration capability when particles are far from the optimal region and enhances exploitation as particles converge. This balance significantly improves convergence speed and solution quality.
2. Delaunay triangulation: Moreover, although Delaunay triangulation has been applied in computational geometry and certain network-related contexts, its integration into a PSO-based WSN coverage optimization framework is novel. This incorporation enables accurate detection and quantification of coverage holes, allowing the optimization process to directly target practical performance metrics such as redundancy reduction and reliability enhancement in WSN deployments.
3. Hammersley Sequence-Based Initialization: We replace conventional random initialization with Hammersley sequence-based initialization (HSI) to ensure a well-distributed initial swarm, reducing the likelihood of premature convergence. Our integration of HSI into VASF-PSO demonstrates for the first time its measurable impact on WSN coverage efficiency.
4. WSN Deployments: The proposed VASF-PSO is evaluated not only on standard benchmark functions but also in real WSN deployment scenarios. We provide a thorough comparison with existing state-of-the-art algorithms (HPO, ABC, IGWO-MS, ICS, and standard PSO), showing superior performance in convergence speed, coverage rate, and node distribution uniformity.

## 3. System Model

### 3.1. Coverage Problem in WSNs

**Figure 2:**
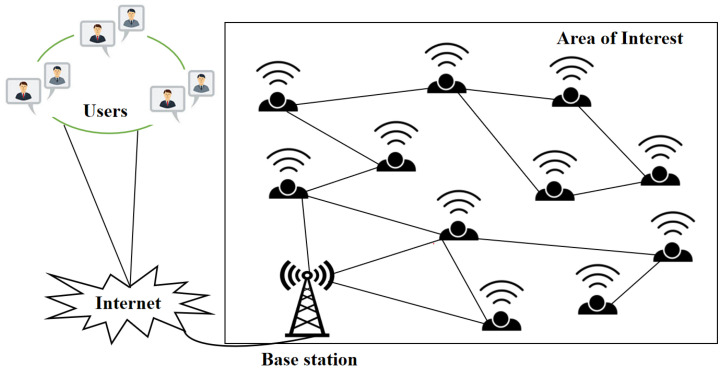
Structure of WSN system.

WSNs encounter a coverage problem that is closely tied to the energy hole issue, where uneven energy consumption among sensor nodes leads to coverage gaps in the monitored area [51]. This happens because certain nodes, especially those near the sink or in high-traffic zones, consume energy faster than others. These nodes, which often relay data for other nodes, deplete their energy more quickly, leading to coverage holes when they stop functioning [52]. This disrupts the network’s primary goal of reliable, continuous data collection. High energy consumption near the sink causes rapid depletion of nearby nodes, isolating distant nodes and reducing overall network performance. The imbalance in energy usage creates localized shortages, affecting coverage. Additionally, in sparse deployments, the failure of a single node can cause significant coverage gaps, while dense deployments may result in excessive energy consumption due to overlapping sensor ranges. These coverage gaps hinder monitoring, compromise data reliability, shorten network lifetime, and can even lead to network partitioning, where nodes beyond the gap lose connectivity to the sink, rendering them ineffective despite having remaining energy [53]. The structure of the WSN network is shown in Figure 2.

Holes are often unavoidable in wireless sensor networks, as they can arise from random node deployment, environmental factors, or external threats [54]. A hole refers to a region that becomes inaccessible or disconnected, either temporarily or permanently, due to node failure, energy depletion, or other disruptions. Such coverage and connectivity gaps can severely impair the network’s sensing and communication capabilities, particularly in critical applications. Prompt detection and remediation are therefore essential to ensure the network’s performance and reliability.

### 3.2. Coverage Problem Optimization Formulation

Sensor nodes are assumed to be randomly distributed within a rectangular region of size $L_{x}\times L_{y}$, and *N* sensor nodes $\left(N_{1},N_{2},N_{3},\ldots ,N_{n}\right)$ are randomly deployed. This random distribution is assumed to be uniform, meaning that the *x* and *y* coordinates of each sensor node are independently drawn from a continuous uniform distribution over the spatial dimensions of the deployment region, i.e., $x∼U(0,L_{x})$ and $y∼U(0,L_{y})$. As a result, each location within the rectangular area has an equal probability of being selected for node placement, ensuring an unbiased and spatially neutral initial deployment. Each node is assigned an equal coverage radius, denoted as $r_{c}$, where $N_{i}=\left(x_{i},y_{i}\right),i=1,2,3,\ldots n$. The research aims to maximize the network’s lifespan while ensuring that the specified coverage requirements have been met by carefully arranging sensor nodes around the region. Following the splitting of the monitoring area into multiple parts, each subregion’s coordinate location is obtained as $R_{j}=\left(x_{j},y_{j},j=1,2,3,\ldots p*q\right)$; following that, the destination point’s Euclidean distance from the sensor node may be expressed as

(1) $$\begin{array}{c} d\left(N_{i},R_{j}\right)=\sqrt{{\left(x_{i}-x_{j}\right)}^{2}+{\left(y_{i}-y_{j}\right)}^{2}}, \end{array}$$

This paper adopts a probabilistic sensing model to more precisely characterize the sensing performance of sensor nodes within the target region, effectively incorporating uncertainties arising from noise and environmental obstacles. In contrast to traditional binary models, where a sensor is assumed to either fully cover a point within a fixed radius or not at all, this model reflects the gradual decline in sensing accuracy as distance from the sensor increases. The sensing probability within the monitored region is thus defined as follows:

(2) $$\begin{array}{c} P\left(N_{i},R_{j}\right)=\left\{\begin{array}{cc} 0, & r_{c}+r_{e}\leq d\left(N_{i},R_{j}\right)\\ e^{-\lambda \alpha^{\beta }}, & r_{c}-r_{e}<d\left(N_{i},R_{j}\right)<R_{s}+R_{u}\\ 1, & r_{c}-r_{e}\geq d\left(N_{i},R_{j}\right), \end{array}\right. \end{array}$$

where $r_{c}$ represents the core sensing radius within which sensing is nearly certain, while $r_{e}$ denotes an extended sensing range beyond the core radius where sensing probability decays exponentially. The term $\alpha =r_{e}-r_{c}+d\left(N_{i},R_{j}\right)$ models the effect of increasing distance on sensing probability. Parameters λ, β, and γ control the rate and shape of this exponential decay, enabling the model to adapt to varying sensor characteristics and environmental conditions.

The total sensing probability, if more than one sensor node can locate the target location, is as follows:

(3) $$\begin{array}{c} P\left(N,R_{j}\right)=1-\prod_{i=1}^{n}\left(1-P\left(N_{i},R_{j}\right)\right), \end{array}$$

where $P\left(N,R_{j}\right)$ is the total sensing probability, *n* represents the total number of sensor nodes, and *N* denotes the set containing all sensors. The objective function for the WSN coverage optimization problem is designed to identify an effective node deployment strategy that maximizes coverage. The coverage rate of a WSN can be characterized as the ratio of the number of targets covered by the sensor set to the total number of targets in the area, expressed as   

(4) $$\begin{array}{c} A_{\mathrm{cov}}=\frac{\sum_{j=1}^{L_{x}\times L_{y}}P\left(N_{i},R_{j}\right)}{L_{x}\times L_{y}}. \end{array}$$

where $A_{\mathrm{cov}}$ represents the coverage rate of the area and is used as the objective function in the WSN coverage optimization.

### 3.3. Conventional Optimization Algorithms

The basic ideas of the conventional PSO algorithm are summarized in this part. In contrast, the following part contains in-depth descriptions of the PSO, ant colony optimization, and simulated annealing (SA) algorithms. In contrast to the conventional PSO, some of these algorithms have been specially modified to deal with the WSN coverage optimization issue. This work compares the basic PSO with other traditional optimization algorithms like ACO, SA, and adaptive particle swarm optimization (APSO), in addition to employing it as the main algorithm. This study emphasizes the benefits of PSO in the context of WSN coverage optimization. PSO is favored for its ability to adapt and be efficient in resolving difficult optimization issues.

#### Traditional PSO

The classic PSO algorithm is a population-based optimization algorithm that draws inspiration from the collective behavior of flocks of birds or schools of fish [55]. A potential solution is represented by each particle in the swarm, which travels around the solution space according to its velocity and the optimal locations discovered by other particles. Since its initial development, PSO has been expanded to address a variety of issues, including discrete, multi-objective, and restricted optimization. It is widely used in fields including biology, engineering, and finance for clustering, parameter estimation, and optimization because of its ease of use and effectiveness. With particles interacting to discover the optimal solution, PSO’s swarm intelligence enables quick exploration and exploitation of the solution space. To ensure quick convergence to the optimum solution, the velocity update method balances exploring the search space with making use of the most prominent position.

PSO iteratively updates the location and velocity of each particle. The velocity modifies the particle’s location by directing movement inside the search space. Until a stopping requirement is satisfied, like a suitable solution, these updates keep coming. In an *N*-dimensional optimization problem, the velocity and location of the *i*th particle are expressed as $V_{it}^{k}=\left(v_{1},v_{2},\ldots ,v_{itN}\right)$ and $X_{it}^{k}=\left(x_{1},x_{2},\ldots ,x_{itN}\right)$. The position and velocity of particle *i* in a swarm of *N* particles are updated as

(5) $$\begin{array}{c} V_{it}^{k}=\omega_{k}V_{it}^{k-1}\;+c_{1}r_{1}\left(P_{best{,}_{it}}\;-X_{i}^{k}\right)\;+\;c_{2}r_{2}\left(G_{best}\;-\;X_{i}^{k}\right), \end{array}$$

(6) $$\begin{array}{c} X_{it}^{k}=X_{it}^{k-1}+V_{it}^{k}. \end{array}$$

The inertial weight is represented by $\omega_{k}$, the optimal location determined by the swarm as a whole is $G_{best}$, whereas $P_{best,it}$ denotes the individual best position for each particle. While $r_{1}$ and $r_{2}$ are random variables falling within the range of [0,1], the acceleration coefficients $c_{1}$ and $c_{2}$ represent the personal and global components, respectively. Furthermore, the particle’s updated velocity is $V_{it}^{k}$, and its current velocity is $V_{it}^{k-1}$. Whereas $X_{it}^{k}$ denotes the itth particle’s prior position, $X_{it}^{k}$ denotes its updated position. In PSO, the acceleration components $c_{1}$ and $c_{2}$ regulate the exploration and exploitation of the search space [56]. The cognitive component, $c_{1}$, determines the impact of the particle’s personal best location $P_{best,it}$ on its velocity update. A larger value of $c_{1}$ leads to a greater influence, pushing the particle to explore its own best. The social component, $c_{2}$, determines the effect of the global best position $G_{best}$ on the particle’s velocity. A higher $c_{2}$ value drives the particle to explore the best position discovered by the swarm. The acceleration components $c_{1}$ and $c_{2}$ are updated as

(7) $$\begin{array}{c} c_{1}^{\left(k\right)}=\left(c_{1f}-c_{1i}\right)\times \frac{i}{iter_{\max}}+c_{1i}, \end{array}$$

(8) $$\begin{array}{c} c_{2}^{\left(k\right)}=\left(c_{2f}-c_{2i}\right)\times \frac{i}{iter_{\max}}+c_{2i}, \end{array}$$

where the term $c_{1}^{\left(k\right)}$ represents the cognitive acceleration coefficient, $c_{2}^{\left(k\right)}$ denotes the social acceleration coefficient, $c_{i}$ and $c_{f}$ refer to the initial and final limits of the acceleration coefficients, respectively.

## 4. Proposed Optimization Algorithm

The PSO algorithm has successfully addressed several deployment issues in WSNs, but improving its fundamental concepts is crucial to its success. Overcoming the drawbacks of the simple PSO algorithm and incorporating more sophisticated algorithms are crucial for achieving better performance in WSN deployment. Due to the dynamic nature of WSN settings, these enhancements can increase convergence speed, resilience, and adaptability while also improving sensor location and energy economy.

### 4.1. Optimization Search Factor

In swarm intelligence (SI) algorithms, such as PSO, finding an appropriate balance between exploration and exploitation is vital. In the PSO algorithm, the balance parameter between exploration and exploitation is the search factor $\omega_{k}$. As $\omega_{k}$ changes dynamically throughout the optimization process, it regulates how much the particle’s past velocity affects its present velocity. A high search factor enables particles to explore a larger portion of the search space, promoting diversity and broad exploration. As the process progresses, particles focus on refining their positions near the best-known solution by reducing the value of $\omega_{k}$ to help exploitation, and the search factor is derived as

(9) $$\begin{array}{c} \omega_{k}=\omega_{\min}+\left(\omega_{\max}-\omega_{\min}\right)\left(1-\frac{|V_{it}^{k-1}|}{V_{\max}}\right). \end{array}$$

where $V_{\max}$ is the maximum allowable velocity for the particle. If the velocity exceeds this value, it is typically clipped to $V_{\max}$ to prevent overlapping behavior in the search space. As the particle’s velocity increases, the search factor $\omega_{k}$ decreases. $\omega_{\min}$ and $\omega_{\max}$ are the minimum and maximum limits of the search factor. The velocity-scaled adaptive search factor $\omega_{k}$ in (9) dynamically adjusts the search factor of each particle according to its velocity magnitude. This approach is motivated by the need to balance exploration and exploitation during the search process. When a particle’s velocity $\left|V_{it}^{k-1}\right|$ is low, it indicates that the particle is potentially near a promising solution region or a local optimum. In this case, increasing the search factor toward $\omega_{\max}$ encourages the particle to explore more broadly and avoid premature convergence. Conversely, when the velocity is high, the search factor is reduced toward $\omega_{\min}$, focusing the search on exploitation to refine the solution around the current position. By normalizing the velocity by its maximum value $V_{\max}$, the search factor is adaptively scaled in each iteration and for each particle, improving convergence speed and preventing stagnation. As the optimization progresses and particle velocities decrease, indicating a trend toward convergence, the adaptive factor reduces the influence of randomness. This results in more refined, localized search behavior, which is essential for fine-tuning node positions to maximize coverage continuity and eliminate minor overlaps and gaps. Like a nonlinear convergence factor, this adaptive mechanism balances exploration and exploitation, facilitating rapid space exploration and precise solution refinement. The pseudo-code of the proposed VASF-PSO algorithm is demonstrated in Algorithm 1.
**Algorithm 1** VASF-PSO pseudocode. 1:**Input:** Initialize swarm size, particle velocities ($V_{i}^{k}$), positions ($X_{i}^{k}$), search factor ($w_{k}$), individual best ($P_{best_{i}}$), swarm best ($G_{best}$), and iteration count. 2:**Output:** Best fitness 3:Initialize particles using Equation (10) 4:Locate the coverage holes using Equations (11)–(13) 5:**for** k = 1 to Max Iterations **do** 6:   **for** i = 1 to swarm size **do** 7:     Calculate the fitness using Equation (4). 8:     **if** fitness <$P_{best_{i}}$ **then** 9:         Update $P_{best_{i}}$ to the current fitness value.10:     **end if**11:     **if** fitness <$G_{best}$ **then**12:         Update $G_{best}$.13:     **end if**14:     Update the velocity of each particle using Equation (5).15:     Update the position of each particle using Equation (6).16:     Calculate acceleration coefficients using Equations (7) and (8)17:     Update the search factor for VASF-PSO using Equation (9)18:     Repeat steps 1 to 18 until the maximum number of iterations is reached.19:   **end for**20:**end for**21:**Return:**$G_{best}$, Optimized continuous WSNs deployment.

To achieve optimal results across various testing configurations, the parameters of the VASF-PSO algorithm are carefully tuned based on empirical testing and sensitivity analysis. The maximum number of iterations *k* is set to 100, and the population size it is selected at 30, falling within the typical range of 20 to 50 particles, considering the problem’s complexity and dimensionality. A significant contribution of the proposed VASF-PSO algorithm is the dynamic adjustment of the search factor $\omega_{k}$, which ranges from $\omega_{\min}=0.2$ to $\omega_{\max}=0.9$. This range was established through extensive empirical testing across various benchmark functions, ensuring stable convergence and a balanced approach between exploration and exploitation in diverse problem landscapes. The cognitive coefficient $c_{1}$ and social coefficient $c_{2}$ are varied using time-varying acceleration coefficients (TVAC), with $c_{1}$ decreasing from 2.5 to 0.5 and $c_{2}$ increasing from 0.5 to 2.5. Initially, a higher $c_{1}$ fosters exploration, while a lower $c_{2}$ reduces the influence of the global best, guiding the search toward the global optimum as iterations progress [57]. To enhance diversity, random numbers $r_{1}$ and $r_{2}$ are initialized within [0, 1], and the velocity range is constrained to [−2, 2]. A moderate value for the velocity limit $V_{\max}$ was empirically selected to facilitate smooth convergence during the optimization process. This careful choice ensures that particles approach optimal solutions gradually, minimizing the risk of overshooting and maintaining the integrity of the search. The search factor range (0.2–0.9) and other parameters in Table 1 were selected based on the standard PSO procedure and validated by initial testing, demonstrating consistent and dependable performance. A comprehensive sensitivity analysis will be part of future work to evaluate how these factors affect the algorithm’s resilience and performance.

### 4.2. Hammersley Sequence-Based Initialization

The Hammersley sequence is a method for generating a set of quasi-random locations that may be used to deploy sensor nodes in WSNs. The purpose of the first sensor node deployment is to guarantee that nodes are distributed uniformly and non-clustered over a region, hence improving network coverage and performance. By distributing sensor nodes evenly throughout the deployment region, the Hammersley sequence avoids gaps or clustering. By reducing the disparity between points, its low-discrepancy behavior improves coverage while using fewer nodes. This leads to increased network coverage, which is necessary for a wireless sensor network to be dependable and effective.

A random distribution is used by the PSO algorithm to initialize the population within the search space. Nevertheless, this algorithm frequently leads to unpredictability, unequal individual distribution, and poor exploration capabilities, all of which may affect the algorithm’s performance. The population was initialized using the Hammersley sequence to get around this limitation. To increase population diversity and speed up the convergence process, the Hammersley sequence is renowned for its uniform distribution qualities, quick convergence, and low computing cost. Particles are dispersed uniformly over the search space due to the Hammersley sequence, which is utilized to initialize the population with a more uniform distribution. The location of each particle *k* is computed by properly scaling the sequence values. A factor *W* is applied to the first coordinate $x_{i}^{\prime }$, and *H* is applied to the second coordinate $y_{i}$, which results in using the Van der Corput sequence with base 2. This method is represented numerically as

(10) $$H_{s}i=\left(x_{i}^{\prime },y_{i}^{\prime }\right)=\left(H_{s}i\times W,\mathrm{VanDerCorput}\left(i,2\right)\times H\right).$$

**Figure 3:**
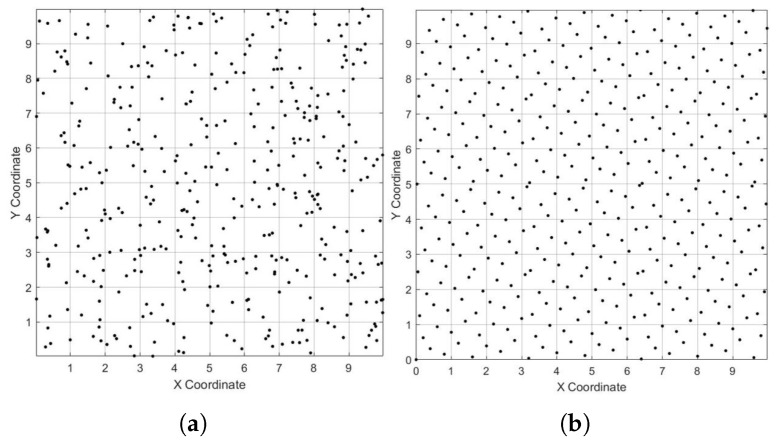
Initial distribution of PSO particles. (**a**) Random initialization. (**b**) Hammersley sequence.

VanDerCorput(i,2) refers to the Van der Corput sequence, which is generated using base 2 for the index *i*. The Van der Corput sequence is a low-discrepancy sequence, producing numbers that are more uniformly distributed compared to random numbers. In the case of WSNs, it ensures that the nodes are spaced more uniformly across the area, especially when dealing with a large number of nodes. The Hammersley sequence is a method for generating a set of quasi-random locations, and random initialization is shown in Figure 3.

### 4.3. Convergence Hole Problem

In this research work, we address the convergence hole problem in WSNs using the Delaunay triangulation method. Delaunay triangulation is an algebraic method that splits a collection of points into independent triangles while ensuring that no point is inside the circumcircle of any triangle. This approach is frequently used in network design to improve coverage and connectivity. The network’s coverage may be optimized via Delaunay triangulation, therefore reducing convergence holes. It ensures that the sensor nodes are evenly distributed across the network, uncovering the convergence hole and leading to more efficient and reliable network operation.

Delaunay triangulation is crucial for identifying coverage holes and network connectivity issues. This method ensures that nodes are connected such that no node is inside the circumcircle of any triangle, thus forming a triangular mesh. Networks with randomly dispersed sensor nodes greatly benefit from this approach as it guarantees that the final network topology minimizes node overlaps and communication delays. Additionally, Delaunay triangulation is crucial for identifying coverage holes and network connectivity issues. By analyzing the connectivity of active nodes, regions with poor connectivity or coverage imbalances can be detected. In particular, large triangles in the mesh may indicate areas where coverage holes exist or where nodes may have exhausted their energy. These gaps can significantly impact the overall performance and coverage of the network. Based on the Delaunay triangulation, the total coverage quality of the network is evaluated by summing the coverage costs across all areas and subtracting the areas of triangles with detected coverage holes, and is numerically presented as

(11) $$D=\sum_{i=1}^{N}\sum_{j=1}^{M}C_{i,j}-\sum_{{▵}_{ijk}\in T}\mathrm{Area}\left(\Delta_{ijk}\right)\cdot ⊮\left(\mathrm{Hole}\;\mathrm{Detected}\right).$$

(12) $$C_{i,j}=\left\{\begin{array}{cc} 1 & \mathrm{if}\;\mathrm{the}\;\mathrm{area}\;\mathrm{at}\;(i,j)\;\mathrm{Area}\;\mathrm{not}\;\mathrm{covered}\;\mathrm{or}\;\mathrm{hole},\\ 0 & \mathrm{if}\;\mathrm{the}\;\mathrm{area}\;\mathrm{at}\;(i,j)\;\mathrm{is}\;\mathrm{covered}. \end{array}\right.$$

where $C_{i,j}$ represents the cost associated with coverage and is defined based on the area of each triangle. The triangle cost is calculated using the area of the triangle formed by nodes $N_{i}$, $N_{j}$, and $N_{k}$, where the area of a triangle with these three nodes is given by

(13) $$\mathrm{Area}\left(\Delta_{ijk}\right)=\frac{1}{2}\left|x_{i}\left(y_{j}-y_{k}\right)+x_{j}\left(y_{k}-y_{i}\right)+x_{k}\left(y_{i}-y_{j}\right)\right|.$$

## 5. Experimental Results and Comparison

The algorithm’s results are assessed in this section, along with localization and WSN coverage tests. The performance of the proposed enhancements is evaluated based on the results obtained from the benchmark functions. In addition, the WSN coverage tests utilize the network coverage model to assess the optimization performance. These tests provide valuable insights into the effectiveness of the proposed changes in improving network coverage and overall algorithm efficiency.

### 5.1. Benchmark Function Tests

#### 5.1.1. Unimodal Benchmark Functions

Unimodal functions were used to assess the proposed algorithm’s quick convergence time since they have unique global optima, which makes them appropriate for this use case. In particular, we evaluated the algorithm’s performance using benchmark functions including Quartic Noise, Sphere, Schwefel 2.22, and Schwefel 2.21. While the Rosenbrock function, often known as the banana function, is a typical test case in optimization studies, the Sphere function, a basic unimodal function, is frequently used to illustrate convergence speed. Because of their difficult landscapes with several local minima, the Schwefel 2.21, 2.22, and 1.2 functions are well-known benchmarks in optimization. As shown in Table 2, these functions are incorporated into our analysis to offer an additional comprehension of the algorithm’s performance.

#### 5.1.2. Multimodal Benchmark Functions

Multimodal functions, on the other hand, are selected to evaluate the algorithm’s capacity to prevent premature convergence since they make finding the global optimum more difficult. Applying VASF-PSO to such functions allowed for the evaluation of its convergence speed and solution consistency. For example, the interaction of its dimensional components makes the Griewank function challenging to optimize. Furthermore, the conventional multimodal penalized functions 1 and 2 make it more difficult to find the global optimum because of their misleading local optima that mimic the global minimum. A non-convex mathematical function, the Rastrigin function has a single global minimum and several local minima, making it a popular benchmark. The Ackley function is another complicated, non-convex function with several local minima and a dramatic peak indicating the global minimum. It is frequently used as a benchmark for algorithms that tackle challenging multimodal optimization issues. Xin-She Yang and Salomon are two more difficult multimodal benchmark functions. According to [58], the search space range for these test functions is carefully chosen to provide a consistent assessment, and the algorithm’s halting conditions are based on a parameter unique to the issue called the threshold. For every benchmark function, the swarm size is 36 and the number of iterations is 1000, where the maximum dimension of the problem space, maximum *d*, is 30 for unimodal and multi-modal benchmark functions and 10 for CEC 2021 benchmark functions. These values are the same for all the variants used for comparison. The Wilcoxon and Friedman tests are used in performance analysis for fully evaluating the optimal outcomes of all algorithms on benchmark functions [59,60]. Table 3 provides the specifics of these multimodal benchmark functions, where the search range represents an area of the search space.

#### 5.1.3. CEC 2021 Benchmark Functions

The algorithms were evaluated with a set of functions that are frequently seen in recent CEC 2021 test suites. For example, the Shifted and fully Rotated Levy function, the Shifted and fully Rotated Expanded Schaffer’s f6 function, and the Shifted and whole Rotated Zakharov function. The algorithm was also tested on various optimization problems using the Hybrid function 2 (N = 6) and the Composition function 4 (N = 6). These test functions thoroughly evaluate the algorithm’s capabilities, spanning various complexity levels and corresponding with the kinds of issues present in the CEC test suites [61]. Table 4 provides the specifics of these CEC 2021 benchmark functions.

### 5.2. Outcome of Related PSO Algorithms

Table 5, Table 6 and Table 7 present the results of a comparison between several variants of PSO across multiple benchmark functions. Six algorithms, traditional PSO [62], linear decreasing inertia weight (LDIW) [63], time-varying acceleration coefficient (TVAC) [57], adaptive weighted delay velocity (AWDV) [64], adaptive weighted PSO (AWPSO) [65], and VASF-PSO, are used to evaluate each function with different dimensions. Table 8 presents the results of a comparison between several variants of PSO across CEC 2021 benchmark functions. For every algorithm, the table presents two performance metrics: Mean, which represents the average solution quality, and standard deviation (SD), which indicates the variation in outcomes. A lower Mean value signifies better optimization performance, while a lower SD indicates greater stability. The findings demonstrate that VASF-PSO consistently achieves the lowest values across most functions, exhibiting superior convergence speed and robustness compared to other methods.

**Figure 4:**
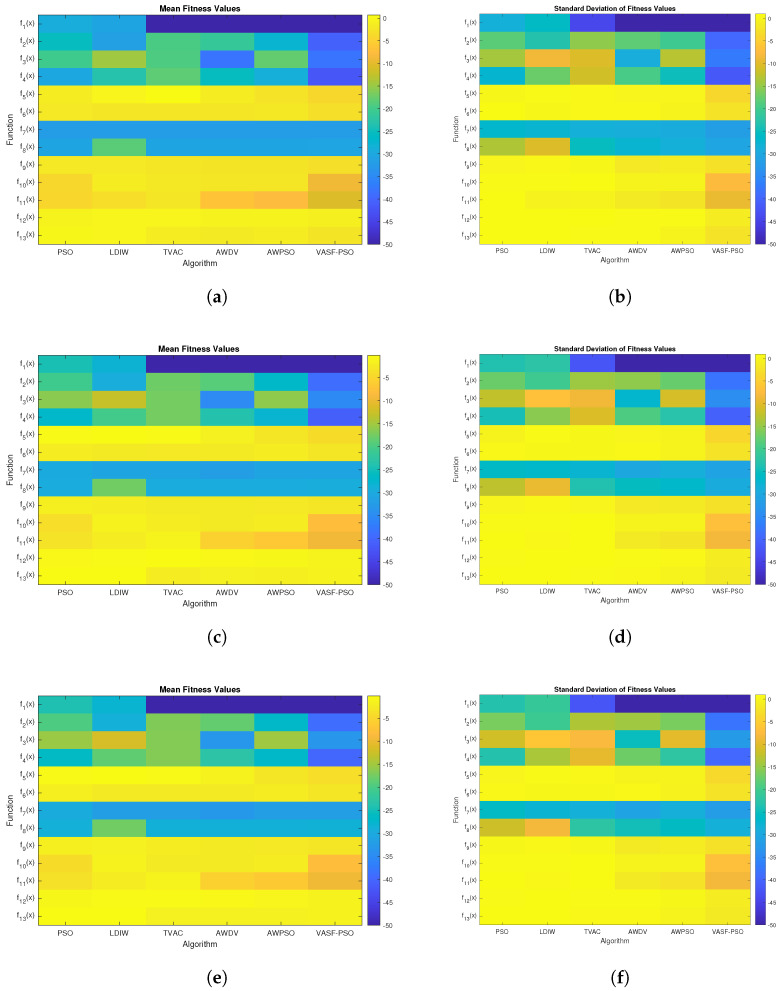
Graphical comparison of mean and standard deviation values corresponding to Table 5, Table 6 and Table 7. (**a**) Table 5 Mean values; (**b**) Table 5 SD values; (**c**) Table 6 Mean values; (**d**) Table 6 SD values; (**e**) Table 7 Mean values; (**f**) Table 7 SD values.

The Friedman test yielded a *p*-value of 1.7026 $\times \;10^{-6}$, which is significantly lower than the commonly used significance level of 0.05 [69]. This strongly indicates that there is a noteworthy difference in the performance of the algorithms, leading us to reject the null hypothesis. As shown in Table 9, the proposed VASF-PSO outperforms all other algorithms, securing the top position with an overall rank of 1. It excels in both unimodal and multimodal functions, consistently achieving first place, thus demonstrating its superior performance compared to the other algorithms. In contrast, PSO performs the worst overall, ranking the lowest among all algorithms, while AWPSO takes second place. Graphical illustrations in Figure 4 complement Table 5, Table 6 and Table 7 by visually representing the comparative data. Heatmaps specifically highlight performance differences and metric distributions across various benchmark functions with different dimensions. Since the VASF-PSO algorithm is tested on minimization benchmark functions, lower values indicate better optimization performance.

Table 10 shows the results of Wilcoxon Signed-Rank tests that compare the VASF-PSO algorithm’s performance to that of five other algorithms: PSO, LDIW, TVAC, AWDV, and AWPSO. For each comparison, the *p*-value represents the probability of significance of the performance difference between VASF-PSO and the other methods. All *p*-values are less than 0.05, indicating that the results are significant. The *p*-value for comparing VASF-PSO with PSO is 0.00073242; for LDIW, it is 0.00024414; for TVAC, it is 0.0012207; for AWDV, it is 0.001709; and for AWPSO, it is 0.0012207. For the CEC 2021 benchmark functions, the proposed VASF-PSO demonstrates competitive and often superior performance compared to other methods. While some comparisons did not yield statistically significant differences, VASF-PSO consistently matched or outperformed the alternatives. Notably, in several cases, especially when compared to the latest PSO variants such as AWDV and AWPSO, the *p*-values approached the significance threshold, proving that VASF-PSO exhibits enhanced effectiveness and robustness in challenging optimization scenarios.

### 5.3. Evaluation of Intelligent Optimization Algorithms

**Figure 5:**
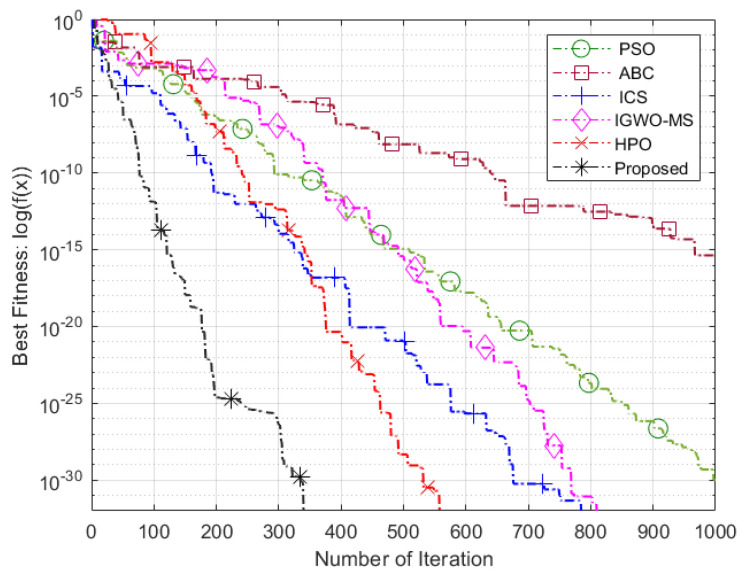
Sphere function.

**Figure 6:**
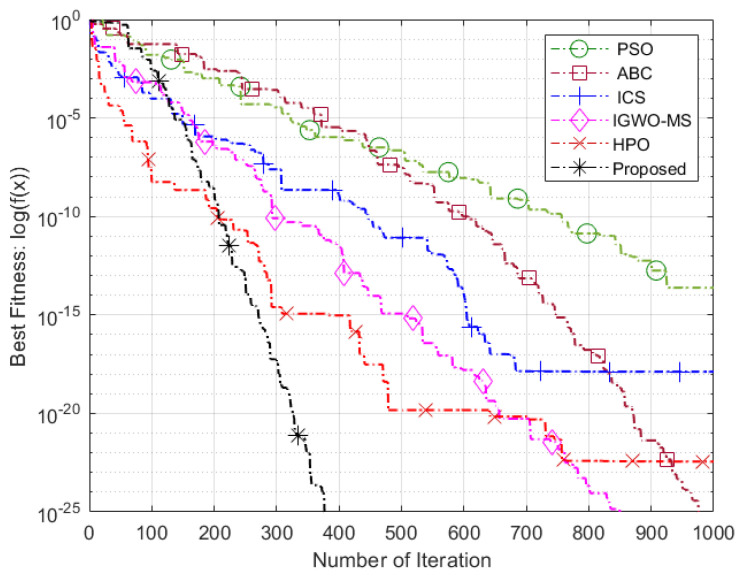
Schwefel 2.21 function.

**Figure 7:**
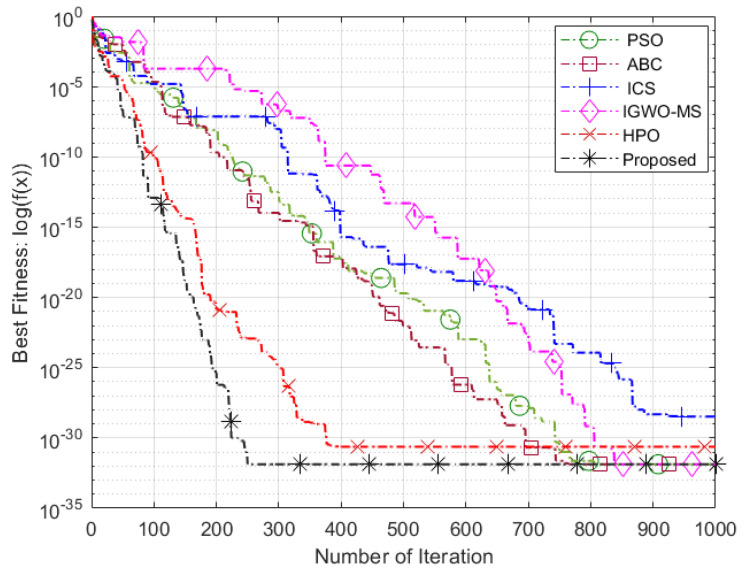
Penalized function.

These benchmark functions’ parameters pose significant challenges for algorithms during the algorithmic optimization procedure. They frequently result in fast convergence toward the nearest optimal value instead of the global optimum, for instance, trapping an algorithm in a local search stagnation situation. Consequently, these functions are perfect for evaluating an algorithm’s ability to overcome local optima problems and keep seeking better solutions. The performance of the proposed algorithm compared to other optimization algorithms on benchmark functions is illustrated in Figure 5, Figure 6 and Figure 7. The proposed algorithm demonstrates the fastest convergence, followed by the HPO algorithm, then IGWO-MS, ICS, ABC, and finally, the PSO algorithm. Using the Hammersley sequence for initialization is responsible for the proposed algorithm’s higher performance. It is commonly known that this sequence produces equally distributed points throughout the search space, ensuring a well-balanced initial population. Because of its uniform distribution qualities, the algorithm can explore more effectively and converge more quickly. Additionally, the computational efficiency of the Hammersley sequence reduces overhead, enhancing the algorithm’s performance and contributing to improved optimization outcomes.

### 5.4. WSN Coverage Performance Optimization

We compared the proposed VASF-PSO algorithm with traditional algorithms like PSO, ABC, ICS, IGWO-MS, and HPO to assess how well it performs in optimizing WSN node deployment while preserving a constant monitoring area and sensor types. The purpose of this comparison was to evaluate VASF-PSO’s performance in enhancing wireless sensor network coverage and optimization.

The proposed algorithm is compared with traditional PSO, ABC, ICS, IGWO-MS, and HPO algorithms. The ABC algorithm, introduced by Karaboga in 2005, is a nature-inspired optimization method that mimics the foraging habits of honeybee colonies [70]. Similar to how bees locate high-quality nectar sources, sensor nodes cooperatively search for and exploit optimal deployment locations, making the ABC method well suited for WSN coverage optimization. Its simplicity and versatility are key advantages, enhancing coverage, energy efficiency, and sensor location optimization in WSNs. Additionally, an improved grey wolf optimizer with multi-strategies is employed to increase deployment efficiency and ensure effective coverage by leveraging the symmetry of sensor node sensing ranges [71]. The efficient implementation of the HPO algorithm in various WSN deployment scenarios is facilitated by its lightweight computational structure. Inspired by predator–prey interactions, this algorithm optimizes WSN coverage by dynamically repositioning sensor nodes (the hunters) to target and cover uncovered or optimal areas (the prey), thus maintaining a balanced exploration–exploitation approach [72]. This algorithm simulates the natural equilibrium where hunters actively seek prey by adjusting their locations relative to the average prey population, focusing on those that are farther away. Furthermore, the improved cuckoo search (ICS) algorithm, inspired by the social behavior of animal groups, is combined with PSO, which updates solutions dimension by dimension, balances exploration and exploitation, and discretizes regional monitoring into point monitoring, ultimately improving coverage in WSNs with a limited number of sensors [73].

For case 1, the monitoring area is 2500 $m^{2}$; for case 2, the monitoring area is 10,000 $m^{2}$; and for case 3, the monitoring area is 22,500 $m^{2}$. In case 1, 32 sensor nodes are used with a sensing range of 5 m; in case 2, 60 sensor nodes are used with a sensing range of 8 m; and in case 3, 36 sensor nodes are used with a sensing range of 15 m.

#### 5.4.1. Case 1

**Figure 8:**
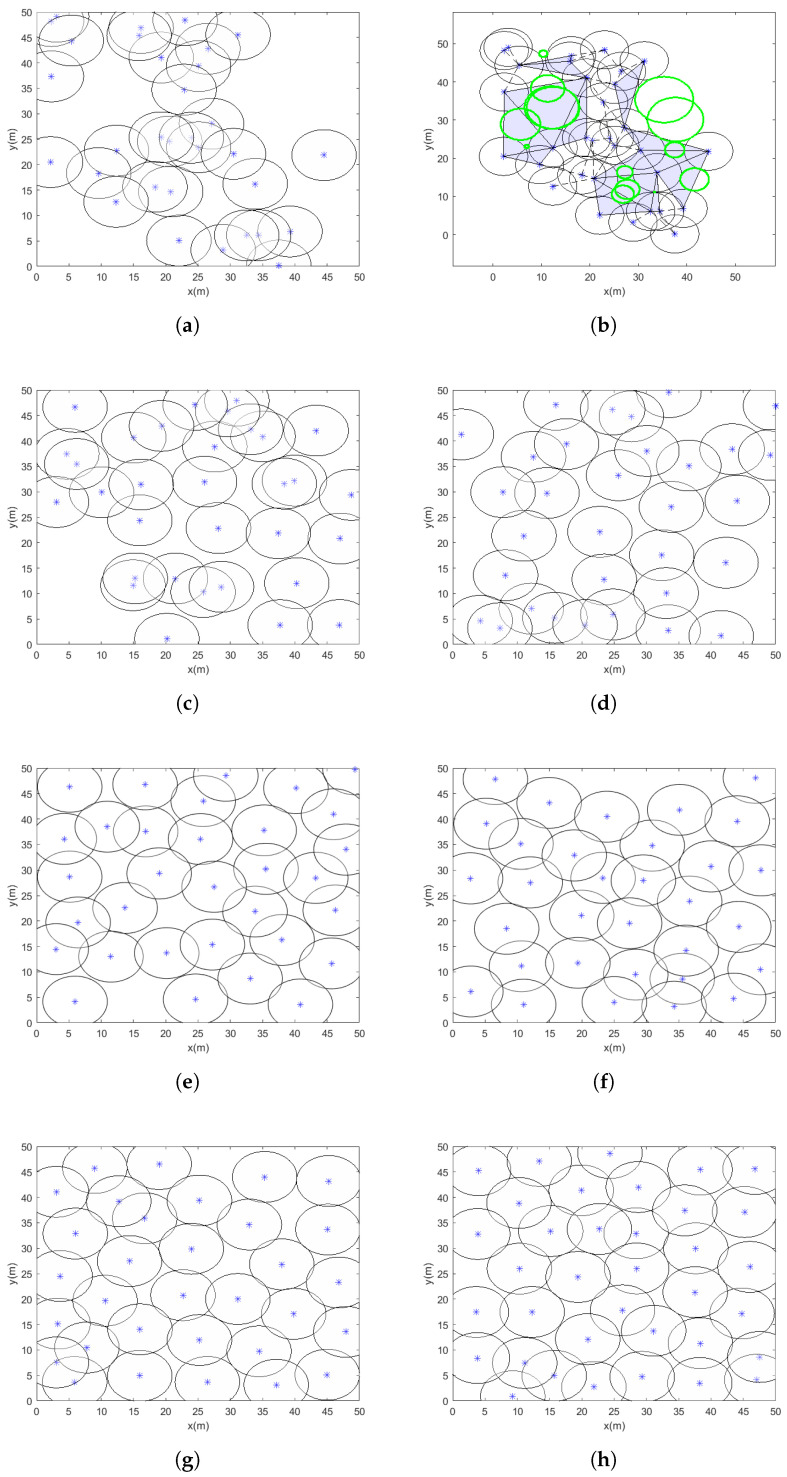
Node deployment case 1: (**a**) the initial deployment of WSNs; (**b**) coverage hole in initial positions of nodes; (**c**) optimized deployment of WSNs by PSO; (**d**) optimized deployment of WSNs by ABC; (**e**) optimized deployment of WSNs by ICS; (**f**) optimized deployment of WSNs by IGWO-MS; (**g**) optimized deployment of WSNs by HPO; (**h**) optimized deployment of WSNs by the proposed algorithm.

Table 11 presents the relevant optimization results for case 1, including the experimental conditions, which feature 32 sensor nodes with a coverage range of 5 m. The radius of the sensor nodes remains constant during the test, with the node placement for case 1 shown in Figure 8, the star is the center of node the black circle is it radius the blue triangle are its coverage gaps and the green circle are covergae hole. Over 20 independent experiments, the proposed VASF-PSO algorithm consistently achieves full coverage in the coverage area for case 1, demonstrating the algorithm’s reliability in optimizing sensor placement. The VASF-PSO algorithm also shows rapid convergence and an efficient optimization process, as it covers the monitored region in fewer than 50 iterations on average.

In contrast, conventional optimization algorithms such as PSO, ABC, ICS, IGWO-MS, and HPO require more than 200 iterations to achieve full coverage, with coverage rates around 90% or lower. In comparison, the VASF-PSO algorithm consistently reaches a 99% coverage rate in fewer than 40 iterations. This significant reduction in the number of iterations highlights the improved efficiency and faster convergence of the VASF-PSO algorithm when compared to traditional algorithms.

#### 5.4.2. Case 2

**Figure 9:**
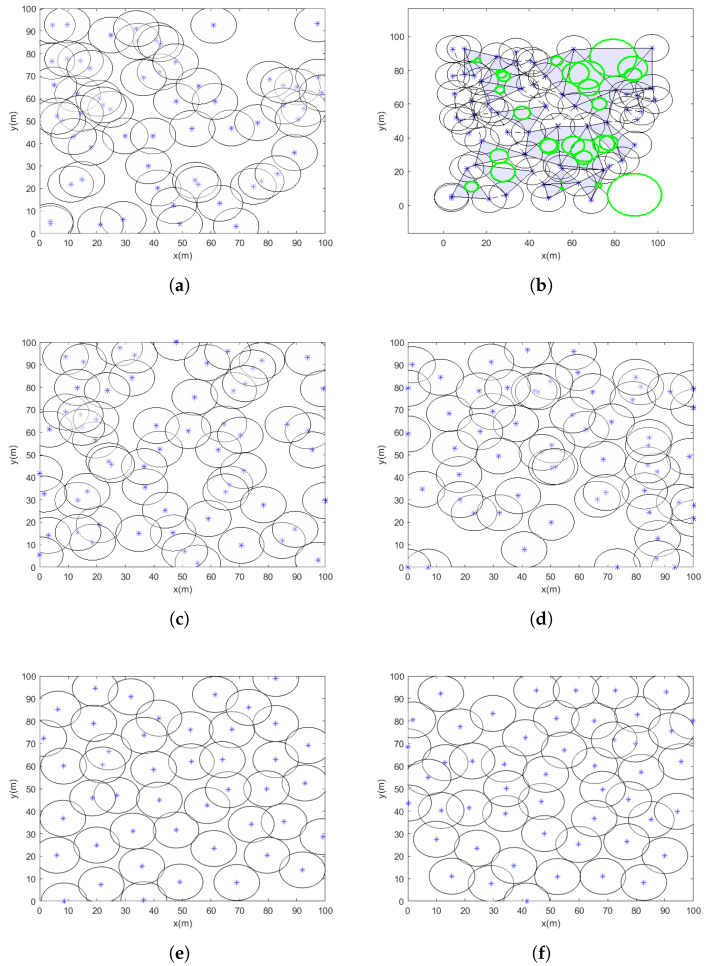

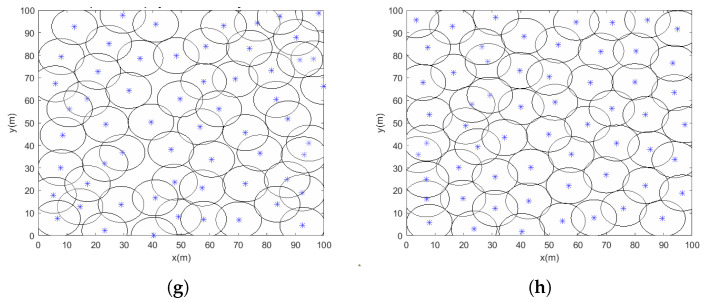
Node deployment case 2: (**a**) the initial deployment of WSNs; (**b**) coverage hole in initial positions of nodes; (**c**) optimized deployment of WSNs by PSO; (**d**) optimized deployment of WSNs by ABC; (**e**) optimized deployment of WSNs by ICS; (**f**) optimized deployment of WSNs by IGWO-MS; (**g**) optimized deployment of WSNs by HPO; (**h**) optimized deployment of WSNs by the proposed algorithm.

In case 2, 60 sensor nodes with an 8 m sensing radius are used in the experiment; the sensor node radius stays constant during the test. In Figure 9, the node arrangement for case 2 is presented. Twenty different tests show that the proposed VASF-PSO algorithm is reliable in maximizing sensor placement, reliably achieving full coverage in the monitored region. The VASF-PSO method also demonstrates quick convergence, completing the region in an average of less than 70 iterations. This efficiency demonstrates how well the method can prevent premature convergence while keeping solution space exploration. From the experiments, we can see the coverage rate, demonstrating the stability and resilience of the optimization strategy. Furthermore, it appears that the algorithm successfully balances node density, preventing redundant overlaps and minimizing coverage holes, based on the uniform distribution of nodes shown in the end deployments.

In comparison, conventional optimization algorithms such as PSO, ABC, ICS, IGWO-MS, and HPO require more than 290 iterations to attain full coverage, with coverage rates around 90% or lower. The VASF-PSO algorithm, on the other hand, consistently achieves 97% to 98% coverage in fewer than 60 iterations, highlighting its superior efficiency and coverage compared to traditional algorithms.

#### 5.4.3. Case 3

**Figure 10:**
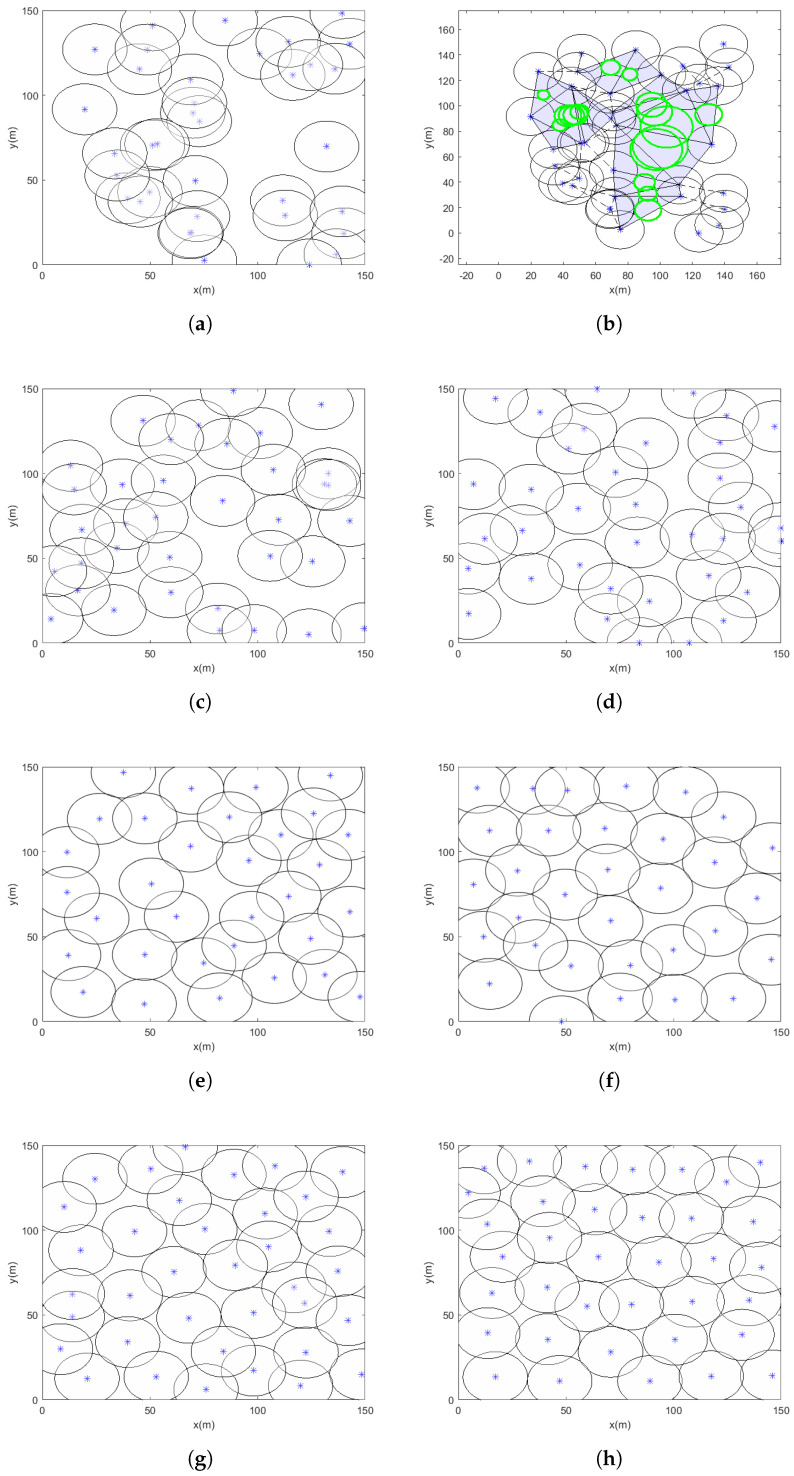
Node deployment case 3: (**a**) the initial deployment of WSNs; (**b**) coverage hole in initial positions of nodes; (**c**) optimized deployment of WSNs by PSO; (**d**) optimized deployment of WSNs by ABC; (**e**) optimized deployment of WSNs by ICS; (**f**) optimized deployment of WSNs by IGWO-MS; (**g**) optimized deployment of WSNs by HPO; (**h**) optimized deployment of WSNs by the proposed algorithm.

The experimental implementation in case 3 consists of 36 sensor nodes, each with a sensing radius of 15 m, which stays fixed for the entire duration of the experiment. Case 3’s sensor node configuration is displayed in Figure 10. The proposed VASF-PSO algorithm continuously achieves full coverage of the monitored region during 20 separate test runs, demonstrating the algorithm’s dependability in carefully positioning sensors for full coverage. The algorithm is capable of finding optimal solutions quickly since it exhibits efficient convergence, covering the full region in an average of less than 85 iterations.

**Figure 11:**
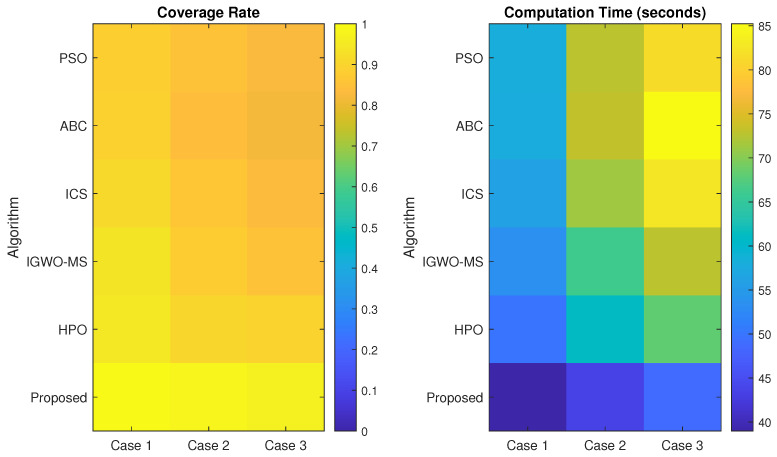
Result visualization of Table 11.

The performance of VASF-PSO is significantly influenced by factors such as node count, node radius, and monitoring area size. Increasing the number of nodes improves coverage but also raises computational costs. Larger node radii result in better coverage, although the benefits begin to diminish beyond a certain point. Expanding the monitoring area typically reduces coverage rates and increases computation time. In contrast, traditional optimization methods like PSO, ABC, ICS, IGWO-MS, and HPO generally achieve coverage rates between 81% and 89% and require over 400 iterations to reach full coverage. In comparison, VASF-PSO consistently achieves a 96% coverage rate in fewer than 85 iterations, demonstrating its superior efficacy in achieving higher coverage with significantly fewer iterations than conventional methods. This illustrates its greater effectiveness and ability to obtain a greater coverage rate in a significantly smaller number of iterations, in contrast to traditional techniques. Table 11 displays the optimization results for cases 1, 2, and 3, which include the coverage area, coverage holes, coverage rate, and total computational time. Graphical illustrations provide additional insight into Figure 11 by visually summarizing simulation results across different coverage scenarios. The figures highlight key performance metrics such as coverage rate and computation time for various algorithms, clearly demonstrating the superior effectiveness and efficiency of the proposed VASF-PSO algorithm under varying area sizes and sensor node configurations.

**Figure 12:**
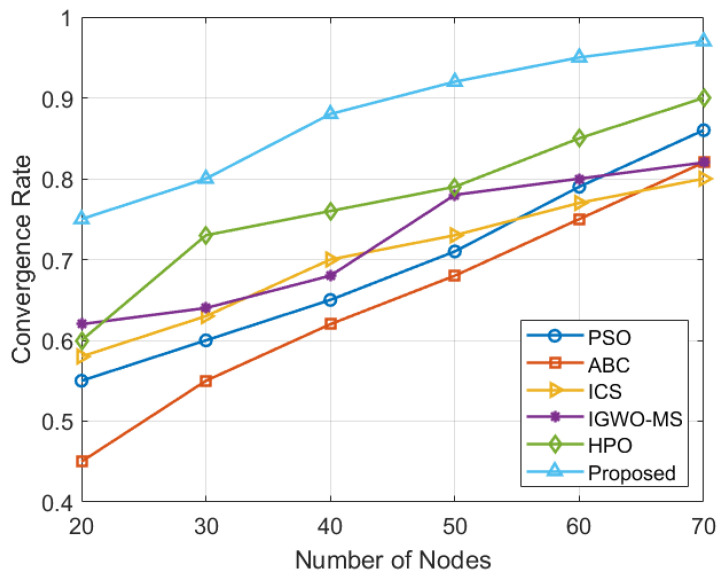
Node density and coverage area.

As shown in Figure 12, when the total number of nodes is small, the solution tends to have a high degree of redundant coverage, meaning that some areas are covered by multiple nodes. However, as the number of nodes increases, the PSO, ABC, ICS, IGWO-MS, and HPO algorithms still show noticeable blind spots in the deployment, indicating that certain regions remain uncovered or inadequately covered, even with the higher node density. In contrast, the VASF-PSO algorithm consistently achieves a higher coverage rate, effectively optimizing node placement and reducing blind spots more efficiently than the other four algorithms across various node density levels.

Comparing the VASF-PSO algorithm to more conventional optimization techniques like PSO, ABC, ICS, IGWO-MS, and HPO, the coverage rate for all three test scenarios is noticeably higher. In particular, case 1 shows a notable increase over traditional techniques, with the VASF-PSO algorithm achieving a 10% higher coverage rate. This increase becomes much more notable in case 2 when VASF-PSO’s superior performance is demonstrated by a 14.71% greater coverage rate. In contrast to conventional algorithms, VASF-PSO further demonstrates its efficacy in enhancing coverage in case 3, where it achieves a 12.94% increase in coverage rate. These results highlight the consistent and substantial advantages of the VASF-PSO algorithm in terms of coverage rate across a variety of scenarios.

## 6. Limitations and Future Work

The VASF-PSO algorithm demonstrates strong coverage performance across varying network scales but also exhibits important limitations. As the number of sensor nodes or the coverage area increases, the computational complexity of the optimization problem grows significantly, resulting in longer processing times and reduced scalability for extensive networks. This increased complexity can challenge real-time or resource-constrained applications. Furthermore, there is an inherent trade-off between maximizing coverage and minimizing energy consumption. Achieving high coverage in larger networks typically requires activating more nodes or increasing node utilization, which leads to higher overall energy expenditure and can significantly impact network lifetime and sustainability. Addressing these challenges through multi-objective optimization frameworks or adaptive energy-aware mechanisms could improve both scalability and energy efficiency. These represent promising directions for future work to enhance the practical applicability of the VASF-PSO algorithm.

## 7. Conclusions

This paper proposes the VASF-PSO algorithm for optimizing sensor node coverage in WSNs. Unlike traditional algorithms, which often struggle with local optima, VASF-PSO enhances coverage performance by improving search accuracy and minimizing the risk of getting stuck in local optima. Key features of the algorithm include Hammersley sequence-based initialization, a velocity-scaled adaptive search factor, and Delaunay triangulation for identifying and filling coverage gaps. Results from three test cases demonstrate the advantages of VASF-PSO. In case 1, with 32 nodes and a 5 m sensing radius, VASF-PSO achieved full coverage in fewer than 50 iterations, while previous algorithms required over 200 iterations. In case 2, with 60 nodes and an 8 m sensing radius, VASF-PSO consistently reached 97–98% coverage in under 60 iterations, compared to just 90% coverage after 290 iterations by conventional algorithms. In case 3, with 36 nodes and a 15 m sensing radius, VASF-PSO achieved 96% coverage in under 85 iterations, while traditional algorithms struggled to reach 89% coverage in over 400 iterations. These results demonstrate that VASF-PSO optimizes coverage more efficiently, requiring fewer iterations and offering improved coverage, outperforming traditional algorithms in WSN coverage optimization. Although VASF-PSO shows promising results, its performance depends on parameter tuning and assumes ideal conditions, without accounting for real-world uncertainties like sensor failures or communication constraints. Future work will focus on improving adaptability in dynamic environments, optimizing energy efficiency, and validating the algorithm through real-world implementations.

## Figures and Tables

**Table 1:** VASF-PSO algorithm parameter selection.

Parameter	Symbol	Value
Population size	it	30
Maximum iterations	*k*	100
Search factor	$\omega_{k}$	0.2–0.9 (Adaptively adjusted by VASF-PSO)
Cognitive coefficient	$c_{1}$	Decreases from 2.5 to 0.5
Social coefficient	$c_{2}$	Increases from 0.5 to 2.5
Velocity range	$V_{\max}$	[−2, 2]
Random numbers	$r_{1},r_{2}$	Random values in [0, 1]

**Table 2:** Unimodal benchmark functions.

Function	Mathematical Expression	Search Space	Max. Velocity	Threshold
Schwefel 2.21	$f_{1}\left(x\right)=\max_{i}\left\{\right|x_{i}\left|,1\leq i\leq 30\right\}$	[−100, 100]	20	0.01
Sphere	$f_{2}\left(x\right)=\sum_{i=1}^{d}{\left(x_{i}\right)}^{2}$	[−100, 100]	20	0.01
Schwefel 2.22	$f_{3}\left(x\right)=\sum_{i=1}^{d}|x_{i}|+\prod_{i=1}^{d}\left|x_{i}\right|$	[−10, 10]	2	0.01
Schwefel 1.2	$f_{4}\left(x\right)=\sum_{i=1}^{d}{\left(\sum_{j=1}^{i}x_{j}\right)}^{2}$	[−100, 100]	20	0.01
Quartic Noise	$f_{5}\left(x\right)=\sum_{i=1}^{d}\left(ix_{i}^{4}+\mathrm{rand}\left[0,1\right)\right)$	[−1.28, 1.28]	0.25	0.1
Rosenbrock	$f_{6}\left(x\right)=\sum_{i=1}^{d-1}\left[100{\left(x_{i+1}-x_{i}^{2}\right)}^{2}+{\left(x_{i}-1\right)}^{2}\right]$	[−30, 30]	6	0.1

**Table 3:** Multimodal benchmark functions.

Function	Mathematical Expression	Search Space	Max. Velocity	Threshold
Penalized 2	$\begin{array}{cc} f_{7}\left(x\right) & =\frac{1}{10}[\sin^{2}\left(3\pi x_{1}\right)+\sum_{i=1}^{d}{\left(x_{i}-1\right)}^{2}\left(1+\sin^{2}\left(3\pi x_{i}+1\right)\right)\\ & \;+{\left(x_{d}-1\right)}^{2}\left(1+\sin\left(2\pi x_{d}\right)\right)+\sum_{i=1}^{d}u\left(x_{i},5,100,4\right)] \end{array}$	[−50,50]	10	0.01
Penalized 1	$\begin{array}{cc} f_{8}\left(x\right) & =\frac{\pi }{n}[10\sin\left(\pi y_{1}\right)+\sum_{i=1}^{d-1}{\left(y_{i}-1\right)}^{2}\left(1+10\sin^{2}\left(\pi y_{i}+1\right)\right)\\ & \;+{\left(y_{d}-1\right)}^{2}+\sum_{i=1}^{d}u\left(x_{i},10,100,4\right)],\\ & y_{i}=1+\frac{x_{i}+1}{4} \end{array}$	[−50,50]	10	0.01
Griewank	$f_{9}\left(x\right)=\sum_{i=1}^{d}\frac{x_{i}^{2}}{4000}-\prod_{i=1}^{d}\cos\left(\frac{x_{i}}{\sqrt{i}}\right)+1$	[−600,600]	120	0.1
Rastrigin	$f_{10}\left(x\right)=10n+\sum_{i=1}^{n}\left(x_{i}^{2}-10\cos\left(2\pi x_{i}\right)\right)$	[−5.12,5.12]	1	0.1
Ackley	$f_{11}\left(x\right)=-ae^{-b\sqrt{\frac{1}{d}\sum x_{i}^{2}}}-e^{\frac{1}{d}\sum \cos\left(cx_{i}\right)}+a+e$	[−32,32]	6.4	0.01
Salomon	$f_{12}\left(x\right)=1-\cos\left(2\pi \sqrt{\sum x_{i}^{2}}\right)+0.1\sqrt{\sum x_{i}^{2}}$	[−20,20]	4	0.1
Xin-She Yang	$f_{13}\left(x\right)=\sum \left|x_{i}\right|e^{-\sum \sin(x_{i}^{2})}$	[−5,5]	1	0.1

**Table 4:** Functions and parameters from the CEC 2021 benchmark set.

No	Function Name	Optimal Value
$f_{14}\left(x\right)$	Shifted and full Rotated Zakharov Function	300
$f_{15}\left(x\right)$	Shifted and full Rotated Expanded Schaffer’s f6	600
$f_{16}\left(x\right)$	Shifted and full Rotated Levy Function	900
$f_{17}\left(x\right)$	Hybrid Function 2 (N = 6)	2000
$f_{18}\left(x\right)$	Composition Function 4 (N = 6)	2700

**Table 5:** Comparison of algorithms across different functions (D = 10).

Function	PSO [66]		LDIW [67]		TVAC [68]		AWDV [64]		AWPSO [65]		VASF-PSO	
	**Mean**	**SD**	**Mean**	**SD**	**Mean**	**SD**	**Mean**	**SD**	**Mean**	**SD**	**Mean**	**SD**
$f_{1}\left(x\right)$	8.61 $\times \;10^{-30}$	4.98 $\times \;10^{-29}$	2.07 $\times \;10^{-31}$	1.69 $\times \;10^{-26}$	6.98 $\times \;10^{-53}$	8.56 $\times \;10^{-45}$	2.76 $\times \;10^{-66}$	6.23 $\times \;10^{-57}$	2.68 $\times \;10^{-72}$	5.34 $\times \;10^{-63}$	6.2 $\times \;10^{-95}$	6.1 $\times \;10^{-94}$
$f_{2}\left(x\right)$	4.83 $\times \;10^{-26}$	5.83 $\times \;10^{-19}$	3.2 $\times \;10^{-32}$	6.23 $\times \;10^{-24}$	3.97 $\times \;10^{-20}$	5.17 $\times \;10^{-16}$	7.50 $\times \;10^{-22}$	7.70 $\times \;10^{-19}$	2.89 $\times \;10^{-28}$	4.96 $\times \;10^{-21}$	2.61 $\times \;10^{-41}$	2.58 $\times \;10^{-40}$
$f_{3}\left(x\right)$	6.56 $\times \;10^{-21}$	7.66 $\times \;10^{-15}$	2.94 $\times \;10^{-15}$	2.94 $\times \;10^{-9}$	5.29 $\times \;10^{-20}$	5.29 $\times \;10^{-11}$	1.18 $\times \;10^{-38}$	1.18 $\times \;10^{-29}$	1.32 $\times \;10^{-18}$	1.32 $\times \;10^{-13}$	8.34 $\times \;10^{-39}$	8.64 $\times \;10^{-38}$
$f_{4}\left(x\right)$	7.43 $\times \;10^{-31}$	7.43 $\times \;10^{-28}$	6.21 $\times \;10^{-24}$	6.21 $\times \;10^{-18}$	5.12 $\times \;10^{-19}$	5.12 $\times \;10^{-12}$	4.06 $\times \;10^{-26}$	4.06 $\times \;10^{-20}$	1.78 $\times \;10^{-29}$	1.78 $\times \;10^{-25}$	1.27 $\times \;10^{-42}$	3.11 $\times \;10^{-42}$
$f_{5}\left(x\right)$	7.05 $\times \;10^{-2}$	1.67	7.67 $\times \;10^{-1}$	5.04	4.78 $\times \;10^{0}$	3.10	3.76 $\times \;10^{-2}$	1.11	2.39 $\times \;10^{-3}$	1.34	1.23 $\times \;10^{-4}$	2.43 $\times \;10^{-4}$
$f_{6}\left(x\right)$	1.7 $\times \;10^{-2}$	7.05	7.0 $\times \;10^{-3}$	2.31	7.6 $\times \;10^{-3}$	2.56	7.6 $\times \;10^{-3}$	2.78	8.3 $\times \;10^{-3}$	9.45 $\times \;10^{-1}$	1.04 $\times \;10^{-3}$	4.89 $\times \;10^{-3}$
$f_{7}\left(x\right)$	1.36 $\times \;10^{-32}$	1.39 $\times \;10^{-27}$	1.39 $\times \;10^{-32}$	3.56 $\times \;10^{-28}$	1.34 $\times \;10^{-32}$	4.78 $\times \;10^{-29}$	1.34 $\times \;10^{-32}$	1.56 $\times \;10^{-29}$	1.34 $\times \;10^{-32}$	4.44 $\times \;10^{-30}$	1.31 $\times \;10^{-32}$	2.45 $\times \;10^{-32}$
$f_{8}\left(x\right)$	2.36 $\times \;10^{-31}$	3.85 $\times \;10^{-14}$	3.85 $\times \;10^{-19}$	6.45 $\times \;10^{-11}$	2.36 $\times \;10^{-31}$	5.56 $\times \;10^{-26}$	2.36 $\times \;10^{-31}$	4.16 $\times \;10^{-28}$	2.30 $\times \;10^{-31}$	3.40 $\times \;10^{-29}$	3.11 $\times \;10^{-31}$	5.12 $\times \;10^{-31}$
$f_{9}\left(x\right)$	1.42 $\times \;10^{-2}$	1.50	1.50 $\times \;10^{-2}$	2.56	7.51 $\times \;10^{-3}$	1.59	4.32 $\times \;10^{-3}$	3.45 $\times \;10^{-2}$	3.54 $\times \;10^{-3}$	8.45 $\times \;10^{-2}$	1.17 $\times \;10^{-3}$	3.39 $\times \;10^{-3}$
$f_{10}\left(x\right)$	1.09 $\times \;10^{-4}$	4.86	4.86 $\times \;10^{-2}$	4.56	8.21 $\times \;10^{-3}$	9.45	5.74 $\times \;10^{-3}$	8.47 $\times \;10^{-1}$	7.34 $\times \;10^{-3}$	8.45 $\times \;10^{-1}$	1.09 $\times \;10^{-9}$	2.67 $\times \;10^{-8}$
$f_{11}\left(x\right)$	5.38 $\times \;10^{-5}$	7.88	6.81 $\times \;10^{-4}$	4.77 $\times \;10^{-1}$	7.66 $\times \;10^{-3}$	8.34 $\times \;10^{-1}$	3.35 $\times \;10^{-7}$	5.57 $\times \;10^{-2}$	2.41 $\times \;10^{-8}$	7.61 $\times \;10^{-3}$	3.17 $\times \;10^{-11}$	4.67 $\times \;10^{-10}$
$f_{12}\left(x\right)$	3.11 $\times \;10^{-1}$	6.34	5.12 $\times \;10^{-1}$	8.25	6.98 $\times \;10^{-1}$	7.88	3.08 $\times \;10^{-1}$	5.98	2.16 $\times \;10^{-1}$	4.34	8.08 $\times \;10^{-2}$	9.98 $\times \;10^{-2}$
$f_{13}\left(x\right)$	8.56 $\times \;10^{-1}$	9.56	7.68 $\times \;10^{-1}$	7.78	3.61 $\times \;10^{-2}$	5.34	2.81 $\times \;10^{-2}$	4.78	3.2 $\times \;10^{-2}$	9.22 $\times \;10^{-1}$	4.23 $\times \;10^{-3}$	5.45 $\times \;10^{-3}$

**Table 6:** Comparison of algorithms across different functions (D = 20).

Function	PSO [66]		LDIW [67]		TVAC [68]		AWDV [64]		AWPSO [65]		VASF-PSO	
	**Mean**	**SD**	**Mean**	**SD**	**Mean**	**SD**	**Mean**	**SD**	**Mean**	**SD**	**Mean**	**SD**
$f_{1}\left(x\right)$	2.85 $\times \;10^{-25}$	3.23 $\times \;10^{-24}$	5.31 $\times \;10^{-29}$	4.14 $\times \;10^{-23}$	2.98 $\times \;10^{-51}$	7.34 $\times \;10^{-43}$	3.08 $\times \;10^{-64}$	7.25 $\times \;10^{-55}$	2.34 $\times \;10^{-70}$	3.55 $\times \;10^{-60}$	4.95 $\times \;10^{-91}$	5.12 $\times \;10^{-90}$
$f_{2}\left(x\right)$	2.03 $\times \;10^{-21}$	4.89 $\times \;10^{-18}$	2.79 $\times \;10^{-30}$	5.11 $\times \;10^{-21}$	1.91 $\times \;10^{-18}$	3.15 $\times \;10^{-15}$	6.56 $\times \;10^{-20}$	6.12 $\times \;10^{-16}$	1.79 $\times \;10^{-27}$	3.12 $\times \;10^{-18}$	1.23 $\times \;10^{-39}$	1.21 $\times \;10^{-38}$
$f_{3}\left(x\right)$	4.92 $\times \;10^{-17}$	6.28 $\times \;10^{-13}$	1.87 $\times \;10^{-13}$	1.54 $\times \;10^{-7}$	4.39 $\times \;10^{-18}$	3.67 $\times \;10^{-9}$	8.01 $\times \;10^{-36}$	8.12 $\times \;10^{-28}$	1.12 $\times \;10^{-16}$	1.42 $\times \;10^{-11}$	5.12 $\times \;10^{-36}$	5.32 $\times \;10^{-35}$
$f_{4}\left(x\right)$	6.31 $\times \;10^{-28}$	6.14 $\times \;10^{-25}$	3.89 $\times \;10^{-21}$	3.68 $\times \;10^{-16}$	4.22 $\times \;10^{-18}$	3.44 $\times \;10^{-11}$	1.29 $\times \;10^{-24}$	1.01 $\times \;10^{-19}$	1.54 $\times \;10^{-28}$	1.44 $\times \;10^{-23}$	1.13 $\times \;10^{-41}$	2.88 $\times \;10^{-41}$
$f_{5}\left(x\right)$	3.67 $\times \;10^{-1}$	0.85	5.49 $\times \;10^{-1}$	3.45	3.21 $\times \;10^{-1}$	2.09	2.59 $\times \;10^{-2}$	0.89	1.15 $\times \;10^{-3}$	0.77	8.39 $\times \;10^{-5}$	1.43 $\times \;10^{-4}$
$f_{6}\left(x\right)$	1.03 $\times \;10^{-2}$	3.25	4.21 $\times \;10^{-3}$	1.87	3.11 $\times \;10^{-3}$	1.98	4.24 $\times \;10^{-3}$	2.44	5.18 $\times \;10^{-3}$	0.65	9.21 $\times \;10^{-4}$	3.56 $\times \;10^{-3}$
$f_{7}\left(x\right)$	9.23 $\times \;10^{-31}$	1.02 $\times \;10^{-26}$	1.04 $\times \;10^{-31}$	2.58 $\times \;10^{-27}$	1.13 $\times \;10^{-31}$	3.21 $\times \;10^{-28}$	1.22 $\times \;10^{-32}$	1.34 $\times \;10^{-30}$	1.06 $\times \;10^{-31}$	2.03 $\times \;10^{-29}$	1.09 $\times \;10^{-31}$	1.51 $\times \;10^{-31}$
$f_{8}\left(x\right)$	1.94 $\times \;10^{-30}$	3.14 $\times \;10^{-13}$	2.99 $\times \;10^{-18}$	5.11 $\times \;10^{-10}$	2.14 $\times \;10^{-30}$	4.55 $\times \;10^{-24}$	2.12 $\times \;10^{-30}$	3.12 $\times \;10^{-26}$	2.18 $\times \;10^{-30}$	2.87 $\times \;10^{-27}$	3.01 $\times \;10^{-30}$	4.79 $\times \;10^{-30}$
$f_{9}\left(x\right)$	1.23 $\times \;10^{-2}$	1.07	1.08 $\times \;10^{-2}$	2.23	4.89 $\times \;10^{-3}$	1.26	3.21 $\times \;10^{-3}$	3.12 $\times \;10^{-2}$	2.13 $\times \;10^{-3}$	5.21 $\times \;10^{-2}$	8.94 $\times \;10^{-4}$	2.89 $\times \;10^{-3}$
$f_{10}\left(x\right)$	9.21 $\times \;10^{-5}$	4.34	4.13 $\times \;10^{-2}$	3.94	5.47 $\times \;10^{-3}$	8.12	4.57 $\times \;10^{-3}$	0.59	6.88 $\times \;10^{-3}$	0.68	7.92 $\times \;10^{-9}$	1.45 $\times \;10^{-7}$
$f_{11}\left(x\right)$	3.88 $\times \;10^{-4}$	6.23	5.93 $\times \;10^{-3}$	3.01	7.01 $\times \;10^{-2}$	6.23	3.01 $\times \;10^{-6}$	0.05	2.58 $\times \;10^{-7}$	5.91 $\times \;10^{-3}$	3.21 $\times \;10^{-10}$	4.13 $\times \;10^{-9}$
$f_{12}\left(x\right)$	2.31 $\times \;10^{-1}$	4.54	4.34 $\times \;10^{-1}$	7.32	5.87 $\times \;10^{-1}$	6.89	2.81 $\times \;10^{-1}$	5.21	1.68 $\times \;10^{-1}$	2.32	5.11 $\times \;10^{-2}$	7.11 $\times \;10^{-2}$
$f_{13}\left(x\right)$	6.93 $\times \;10^{-1}$	8.54	6.23 $\times \;10^{-1}$	6.44	1.22 $\times \;10^{-2}$	3.56	2.32 $\times \;10^{-2}$	3.01	1.23 $\times \;10^{-2}$	0.80	3.79 $\times \;10^{-2}$	4.01 $\times \;10^{-2}$

**Table 7:** Comparison of algorithms across different functions (D = 30).

Function	PSO [66]		LDIW [67]		TVAC [68]		AWDV [64]		AWPSO [65]		VASF-PSO	
	**Mean**	**SD**	**Mean**	**SD**	**Mean**	**SD**	**Mean**	**SD**	**Mean**	**SD**	**Mean**	**SD**
$f_{1}\left(x\right)$	5.93 $\times \;10^{-25}$	6.45 $\times \;10^{-24}$	1.34 $\times \;10^{-28}$	9.74 $\times \;10^{-22}$	4.57 $\times \;10^{-51}$	6.12 $\times \;10^{-43}$	4.78 $\times \;10^{-62}$	8.03 $\times \;10^{-54}$	3.21 $\times \;10^{-68}$	5.47 $\times \;10^{-57}$	6.22 $\times \;10^{-90}$	6.58 $\times \;10^{-89}$
$f_{2}\left(x\right)$	1.93 $\times \;10^{-20}$	3.45 $\times \;10^{-17}$	1.65 $\times \;10^{-29}$	3.45 $\times \;10^{-21}$	1.78 $\times \;10^{-17}$	2.98 $\times \;10^{-14}$	5.23 $\times \;10^{-19}$	5.89 $\times \;10^{-15}$	1.22 $\times \;10^{-27}$	3.21 $\times \;10^{-17}$	1.16 $\times \;10^{-39}$	1.14 $\times \;10^{-38}$
$f_{3}\left(x\right)$	3.27 $\times \;10^{-16}$	4.12 $\times \;10^{-12}$	1.67 $\times \;10^{-12}$	1.12 $\times \;10^{-6}$	3.12 $\times \;10^{-17}$	2.34 $\times \;10^{-8}$	7.68 $\times \;10^{-34}$	7.43 $\times \;10^{-26}$	1.14 $\times \;10^{-15}$	1.21 $\times \;10^{-10}$	5.61 $\times \;10^{-34}$	5.85 $\times \;10^{-33}$
$f_{4}\left(x\right)$	4.72 $\times \;10^{-27}$	5.81 $\times \;10^{-24}$	2.58 $\times \;10^{-19}$	2.34 $\times \;10^{-14}$	3.14 $\times \;10^{-17}$	2.56 $\times \;10^{-10}$	1.06 $\times \;10^{-23}$	8.97 $\times \;10^{-18}$	1.23 $\times \;10^{-27}$	1.12 $\times \;10^{-22}$	1.09 $\times \;10^{-40}$	2.76 $\times \;10^{-40}$
$f_{5}\left(x\right)$	4.09 $\times \;10^{-1}$	1.22	4.88 $\times \;10^{-1}$	3.67	4.22 $\times \;10^{-1}$	2.65	3.45 $\times \;10^{-2}$	0.72	1.32 $\times \;10^{-3}$	0.62	1.15 $\times \;10^{-4}$	2.56 $\times \;10^{-4}$
$f_{6}\left(x\right)$	1.31 $\times \;10^{-2}$	2.99	5.23 $\times \;10^{-3}$	1.43	4.09 $\times \;10^{-3}$	1.97	5.18 $\times \;10^{-3}$	2.23	6.11 $\times \;10^{-3}$	5.34 $\times \;10^{-1}$	1.32 $\times \;10^{-3}$	4.56 $\times \;10^{-3}$
$f_{7}\left(x\right)$	1.34 $\times \;10^{-30}$	1.29 $\times \;10^{-26}$	1.02 $\times \;10^{-32}$	2.43 $\times \;10^{-28}$	1.21 $\times \;10^{-32}$	3.76 $\times \;10^{-29}$	1.15 $\times \;10^{-33}$	1.67 $\times \;10^{-31}$	1.28 $\times \;10^{-32}$	2.89 $\times \;10^{-29}$	1.12 $\times \;10^{-32}$	1.56 $\times \;10^{-32}$
$f_{8}\left(x\right)$	1.56 $\times \;10^{-29}$	3.21 $\times \;10^{-12}$	3.12 $\times \;10^{-18}$	5.12 $\times \;10^{-9}$	2.04 $\times \;10^{-29}$	5.66 $\times \;10^{-23}$	2.02 $\times \;10^{-29}$	4.56 $\times \;10^{-25}$	2.19 $\times \;10^{-29}$	2.94 $\times \;10^{-26}$	3.12 $\times \;10^{-29}$	4.34 $\times \;10^{-29}$
$f_{9}\left(x\right)$	1.98 $\times \;10^{-2}$	1.56	2.56 $\times \;10^{-2}$	3.12	6.72 $\times \;10^{-3}$	1.68	3.89 $\times \;10^{-3}$	3.56 $\times \;10^{-2}$	3.01 $\times \;10^{-3}$	7.61 $\times \;10^{-2}$	1.23 $\times \;10^{-3}$	3.01 $\times \;10^{-3}$
$f_{10}\left(x\right)$	8.76 $\times \;10^{-5}$	4.12	3.68 $\times \;10^{-2}$	3.43	4.67 $\times \;10^{-3}$	7.89	4.25 $\times \;10^{-3}$	6.12 $\times \;10^{-1}$	5.97 $\times \;10^{-3}$	6.01 $\times \;10^{-1}$	8.76 $\times \;10^{-9}$	1.23 $\times \;10^{-7}$
$f_{11}\left(x\right)$	2.45 $\times \;10^{-4}$	5.76	5.29 $\times \;10^{-3}$	2.89	7.21 $\times \;10^{-2}$	5.67	2.76 $\times \;10^{-6}$	4.45 $\times \;10^{-2}$	2.98 $\times \;10^{-7}$	5.32 $\times \;10^{-3}$	2.12 $\times \;10^{-10}$	4.76 $\times \;10^{-9}$
$f_{12}\left(x\right)$	1.56 $\times \;10^{-1}$	3.78	3.12 $\times \;10^{-1}$	5.67	4.67 $\times \;10^{-1}$	5.01	2.19 $\times \;10^{-1}$	4.78	1.12 $\times \;10^{-1}$	1.88	4.56 $\times \;10^{-2}$	6.56 $\times \;10^{-2}$
$f_{13}\left(x\right)$	7.34 $\times \;10^{-1}$	8.56	6.87 $\times \;10^{-1}$	6.21	2.56 $\times \;10^{-2}$	3.12	2.12 $\times \;10^{-2}$	2.45	2.31 $\times \;10^{-2}$	7.12 $\times \;10^{-1}$	3.92 $\times \;10^{-2}$	4.76 $\times \;10^{-2}$

**Table 8:** Selected CEC 2021 benchmark functions (D = 10).

Function	PSO [66]		LDIW [67]		TVAC [68]		AWDV [64]		AWPSO [65]		VASF-PSO	
	**Mean**	**SD**	**Mean**	**SD**	**Mean**	**SD**	**Mean**	**SD**	**Mean**	**SD**	**Mean**	**SD**
$f_{14}\left(x\right)$	3.00 $\times \;10^{2}$	4.44 $\times \;10^{-5}$	7.71 $\times \;10^{3}$	1.11 $\times \;10^{3}$	6.66 $\times \;10^{2}$	8.49 $\times \;10^{2}$	5.45 $\times \;10^{2}$	6.78 $\times \;10^{3}$	5.04 $\times \;10^{2}$	6.21 $\times \;10^{3}$	3.20 $\times \;10^{2}$	3.11 $\times \;10^{2}$
$f_{15}\left(x\right)$	6.02 $\times \;10^{2}$	3.35 $\times \;10^{1}$	6.00 $\times \;10^{2}$	7.8 $\times \;10^{-1}$	6.27 $\times \;10^{2}$	6.19 $\times \;10^{2}$	6.10 $\times \;10^{2}$	7.70 $\times \;10^{2}$	5.80 $\times \;10^{2}$	6.66 $\times \;10^{2}$	1.88 $\times \;10^{1}$	4.99 $\times \;10^{0}$
$f_{16}\left(x\right)$	9.00 $\times \;10^{2}$	2.73 $\times \;10^{-2}$	9.01 $\times \;10^{2}$	7.35 $\times \;10^{-1}$	4.33 $\times \;10^{3}$	9.68 $\times \;10^{2}$	5.45 $\times \;10^{2}$	6.45 $\times \;10^{3}$	3.44 $\times \;10^{2}$	6.45 $\times \;10^{3}$	3.14 $\times \;10^{2}$	3.74 $\times \;10^{1}$
$f_{17}\left(x\right)$	2.02 $\times \;10^{3}$	1.22 $\times \;10^{2}$	2.20 $\times \;10^{3}$	2.01 $\times \;10^{-2}$	1.64 $\times \;10^{3}$	2.23 $\times \;10^{2}$	3.56 $\times \;10^{3}$	4.34 $\times \;10^{1}$	3.45 $\times \;10^{3}$	2.64 $\times \;10^{1}$	2.02 $\times \;10^{3}$	8.86 $\times \;10^{0}$
$f_{18}\left(x\right)$	2.86 $\times \;10^{3}$	2.59 $\times \;10^{1}$	2.86 $\times \;10^{3}$	2.06 $\times \;10^{-1}$	2.88 $\times \;10^{3}$	2.20 $\times \;10^{2}$	2.92 $\times \;10^{3}$	2.15 $\times \;10^{1}$	2.93 $\times \;10^{3}$	2.08 $\times \;10^{2}$	2.86 $\times \;10^{3}$	1.13 $\times \;10^{0}$

**Table 9:** Friedman test results and algorithm rankings.

Algorithm	Unimodal Average Rank	Unimodal Overall Rank	Multimodal Average Rank	Multimodal Overall Rank	Overall Average Rank	Overall Rank
VASF-PSO	2.29	1	2.00	1	2.15	1
AWPSO	2.43	2	3.33	2	2.85	2
AWDV	2.86	3	3.00	4	2.92	3
LDIW	3.57	5	3.00	3	3.31	4
TVAC	3.86	4	3.83	6	3.85	5
PSO	6.00	6	5.83	5	5.92	6

**Table 10:** Wilcoxon test significant comparisons.

Comparison	Conclusion
VASF-PSO vs. PSO	VASF-PSO > PSO (*p* = 0.00073242)
VASF-PSO vs. LDIW	VASF-PSO > LDIW (*p* = 0.00024414)
VASF-PSO vs. TVAC	VASF-PSO > TVAC (*p* = 0.0012207)
VASF-PSO vs. AWDV	VASF-PSO > AWDV (*p* = 0.001709)
VASF-PSO vs. AWPSO	VASF-PSO > AWPSO (*p* = 0.0012207)

**Table 11:** Simulation result analysis.

Parameters (m)			
	Case 1	Case 2	Case 3
Coverage Area	[50, 50]	[100, 100]	[150, 150]
Sensor Nodes	32	60	36
Sensing Radius	5 m	8 m	15 m
Coverage Holes	11	23	15
Coverage Rate			
Algorithm	Case 1	Case 2	Case 3
PSO	0.8856	0.8545	0.8278
ABC	0.8945	0.8434	0.8195
ICS	0.9105	0.8656	0.8345
IGWO-MS	0.9387	0.8813	0.8534
HPO	0.9456	0.9034	0.8967
Proposed	0.9945	0.9810	0.9690
Computation Time (s)			
Algorithm	Case 1	Case 2	Case 3
PSO	58.09	72.81	81.56
ABC	57.78	73.18	85.23
ICS	56.11	71.45	82.67
IGWO-MS	53.45	66.25	72.78
HPO	49.89	61.10	68.19
Proposed	38.98	43.56	48.88

## Data Availability

Data are contained within this article.

## Abbreviations

The following abbreviations are used in this manuscript:

PSO	Particle swarm optimization
WSNs	Wireless sensor networks
VASF-PSO	Velocity-scaled adaptive search factor PSO
HPO	Hunter–prey optimization
IGWO-MS	Improved grey wolf optimizer with multi-strategies
ICS	Improved cuckoo search
ABC	Artificial bee colony
QoS	Quality of service
BS	Base station
SNs	Sensor nodes

## References

[1] Oubbati O.S., Atiquzzaman M., Lim H., Rachedi A., Lakas A. (2022). Synchronizing UAV teams for timely data collection and energy transfer by deep reinforcement learning. IEEE Trans. Veh. Technol..

[2] Fascista A. (2022). Toward integrated large-scale environmental monitoring using WSN/UAV/Crowdsensing: A review of applications, signal processing, and future perspectives. Sensors.

[3] Bokhari M.U., Kareem A., Hanafi B. (2024). Exploring Fault Tolerance Consensus for Wireless Sensor Networks: A Comprehensive Detailed Study. J. Electr. Syst..

[4] Sheng Z., Mahapatra C., Zhu C., Leung V.C. (2015). Recent advances in industrial wireless sensor networks toward efficient management in IoT. IEEE Access.

[5] Coboi A., Nguyen M.T., Primeiro I.Z., Van Nam P., Van Huy B., Ta T.M., Nguyen T.T. (2024). Towards Multiple Sources for Energy Harvesting in Wireless Sensor Networks in Practical Applications. Comput. Netw. Commun..

[6] Xia Y., Deng X., Yi L., Liu S., Yang L.T., Zhu C., Tang X. (2023). A trust-based reliable confident information coverage model of wireless sensor networks for intelligent transportation. IEEE Trans. Veh. Technol..

[7] Heath R.W., Kountouris M., Bai T. (2013). Modeling heterogeneous network interference using Poisson point processes. IEEE Trans. Signal Process..

[8] Shah S.L., Abbas Z.H., Abbas G., Muhammad F., Hussien A., Baker T. (2023). An innovative clustering hierarchical protocol for data collection from remote wireless sensor networks based internet of things applications. Sensors.

[9] Lima M.M., Sardinha E.D., Balico L.N., Oliveira H.A. (2023). PAtCH: Proactive approach to circumvent holes in wireless sensor networks. Sensors.

[10] Gola K.K., Gupta B. (2019). An energy-efficient quality of service (QOS) parameter-based void avoidance routing technique for underwater sensor networks. Jordanian J. Comput. Inf. Technol..

[11] Guleria K., Verma A.K. (2019). Comprehensive review for energy efficient hierarchical routing protocols on wireless sensor networks. Wirel. Netw..

[12] Ma D., Lan G., Hassan M., Hu W., Das S.K. (2019). Sensing, computing, and communications for energy harvesting IoTs: A survey. IEEE Commun. Surv. Tutor..

[13] Majid M., Habib S., Javed A.R., Rizwan M., Srivastava G., Gadekallu T.R., Lin J.C.W. (2022). Applications of wireless sensor networks and internet of things frameworks in the industry revolution 4.0: A systematic literature review. Sensors.

[14] Ray P.P., Mukherjee M., Shu L. (2017). Internet of Things for Disaster Management: State-of-the-Art and Prospects. IEEE Access.

[15] Li K., Voicu R.C., Kanhere S.S., Ni W., Tovar E. (2019). Energy Efficient Legitimate Wireless Surveillance of UAV Communications. IEEE Trans. Veh. Technol..

[16] Kang J.J., Yang W., Dermody G., Ghasemian M., Adibi S., Haskell-Dowland P. (2020). No Soldiers Left Behind: An IoT-Based Low-Power Military Mobile Health System Design. IEEE Access.

[17] Osanaiye O.A., Alfa A.S., Hancke G.P. (2018). Denial of Service Defence for Resource Availability in Wireless Sensor Networks. IEEE Access.

[18] Raza M., Aslam N., Le-Minh H., Hussain S., Cao Y., Khan N.M. (2018). A Critical Analysis of Research Potential, Challenges, and Future Directives in Industrial Wireless Sensor Networks. IEEE Commun. Surv. Tutor..

[19] Jamshed M.A., Ali K., Abbasi Q.H., Imran M.A., Ur-Rehman M. (2022). Challenges, applications, and future of wireless sensors in Internet of Things: A review. IEEE Sens. J..

[20] Al-Karaki J.N., Gawanmeh A. (2017). The optimal deployment, coverage, and connectivity problems in wireless sensor networks: Revisited. IEEE Access.

[21] Zhang Z., Mehmood A., Shu L., Huo Z., Zhang Y., Mukherjee M. (2018). A survey on fault diagnosis in wireless sensor networks. IEEE Access.

[22] Latif K., Javaid N., Ahmad A., Khan Z.A., Alrajeh N., Khan M.I. (2016). On energy hole and coverage hole avoidance in underwater wireless sensor networks. IEEE Sens. J..

[23] Ekhlas V.R., Shirvani M.H., Dana A., Raeisi N. (2023). Discrete grey wolf optimization algorithm for solving k-coverage problem in directional sensor networks with network lifetime maximization viewpoint. Appl. Soft Comput..

[24] Shi Y., Yang K., Jiang T., Zhang J., Letaief K.B. (2020). Communication-efficient edge AI: Algorithms and systems. IEEE Commun. Surv. Tutor..

[25] Li J., Yang L., Wu Q., Lei X., Zhou F., Shu F., Mu X., Liu Y., Fan P. (2024). Active RIS-aided NOMA-enabled space-air-ground integrated networks with cognitive radio. IEEE J. Sel. Areas Commun..

[26] Sharma V., Patel R., Bhadauria H.S., Prasad D. (2016). NADS: Neighbor assisted deployment scheme for optimal placement of sensor nodes to achieve blanket coverage in wireless sensor network. Wirel. Pers. Commun..

[27] Pandey D., Kushwaha V. (2024). An Exploratory Study of Optimization Techniques for Congestion Control in Wireless Sensor Networks. Adhoc Sens. Wirel. Netw..

[28] Vu H.N., Pham C., Dung N.M., Ro S. (2020). Detecting and tracking sinkholes using multi-level convolutional neural networks and data association. IEEE Access.

[29] Guo J., Sun Y., Liu T., Li Y., Fei T. (2025). An Optimization Coverage Strategy for Wireless Sensor Network Nodes Based on Path Loss and False Alarm Probability. Sensors.

[30] Liu Y., Li Q. (2023). Coverage algorithm based on perceived environment around nodes in mobile wireless sensor networks. Wirel. Pers. Commun..

[31] Neggaz N., Seyyedabbasi A., Hussien A.G., Rahim M., Beşkirli M. (2024). Optimal nodes localization in wireless sensor networks using Nutcracker optimizer algorithms: Istanbul Area. IEEE Access.

[32] Mishra S.D., Verma D. (2024). Energy-efficient and reliable clustering with optimized scheduling and routing for wireless sensor networks. Multimed. Tools Appl..

[33] Yue Y.G., He P. (2018). A comprehensive survey on the reliability of mobile wireless sensor networks: Taxonomy, challenges, and future directions. Inf. Fusion.

[34] Ahmed M.M., Houssein E.H., Hassanien A.E., Taha A., Hassanien E. (2018). Maximizing lifetime of wireless sensor networks based on whale optimization algorithm. Proceedings of the International Conference on Advanced Intelligent Systems and Informatics.

[35] Lakshmi Y.V., Singh P., Abouhawwash M., Mahajan S., Pandit A.K., Ahmed A.B. (2022). Improved Chan algorithm based optimum UWB sensor node localization using hybrid particle swarm optimization. IEEE Access.

[36] Wang M., Wang X., Jiang K., Fan B. (2022). Reinforcement learning-enabled resampling particle swarm optimization for sensor relocation in reconfigurable WSNs. IEEE Sens. J..

[37] Zhang Y., Shen W. (2021). A novel particle swarm optimization algorithm for k-coverage problems in wireless sensor networks. Proceedings of the 2021 IEEE 24th International Conference on Computer Supported Cooperative Work in Design (CSCWD).

[38] Cao L., Yue Y., Cai Y., Zhang Y. (2021). A novel coverage optimization strategy for heterogeneous wireless sensor networks based on connectivity and reliability. IEEE Access.

[39] Yao Y., Liao H., Liu M., Yang X. (2023). Coverage optimization strategy for 3-D wireless sensor networks based on improved sparrow search algorithm. IEEE Sens. J..

[40] Zhao Q., Li C., Zhu D., Xie C. (2022). Coverage optimization of wireless sensor networks using combinations of PSO and chaos optimization. Electronics.

[41] Gamal M., Mekky N.E., Soliman H., Hikal N.A. (2022). Enhancing the lifetime of wireless sensor networks using fuzzy logic LEACH technique-based particle swarm optimization. IEEE Access.

[42] Haris M., Bhatti D.M.S., Nam H. (2024). A Fast-Convergent Hyperbolic Tangent PSO Algorithm for UAVs Path Planning. IEEE Open J. Veh. Technol..

[43] Li D., Wen X.B. (2015). An Improved PSO Algorithm for Distributed Localization in Wireless Sensor Networks. Int. J. Distrib. Sens. Netw..

[44] Meng Y., Zhi Q., Zhang Q., Yao N. (2020). A Two-Stage Particle Swarm Optimization Algorithm for Wireless Sensor Nodes Localization in Concave Regions. Information.

[45] Kim Y.G., Lee M.J. (2014). Scheduling multi-channel and multi-timeslot in time-constrained wireless sensor networks via simulated annealing and particle swarm optimization. IEEE Commun. Mag..

[46] Gupta N., Kumar N., Jain S. Coverage problem in wireless sensor networks: A survey. Proceedings of the 2016 International Conference on Signal Processing, Communication, Power and Embedded System (SCOPES).

[47] Amutha J., Sharma S., Sharma S.K. (2021). Strategies based on various aspects of clustering in wireless sensor networks using classical, optimization and machine learning techniques: Review, taxonomy, research findings, challenges and future directions. Comput. Sci. Rev..

[48] Sheng Z., Mahapatra C., Leung V.C., Chen M., Sahu P.K. (2015). Energy efficient cooperative computing in mobile wireless sensor networks. IEEE Trans. Cloud Comput..

[49] Hacioglu G., Kand V.F.A., Sesli E. (2016). Multi objective clustering for wireless sensor networks. Expert Syst. Appl..

[50] Elshakhs Y.S., Deliparaschos K.M., Charalambous T., Oliva G., Zolotas A. (2024). A comprehensive survey on Delaunay triangulation: Applications, algorithms, and implementations over CPUs, GPUs, and FPGAs. IEEE Access.

[51] Thomas D., Shankaran R., Sheng Q.Z., Orgun M.A., Hitchens M., Masud M., Ni W., Mukhopadhyay S.C., Piran M.J. (2020). QoS-aware energy management and node scheduling schemes for sensor network-based surveillance applications. IEEE Access.

[52] Samadi R., Nazari A., Seitz J. (2023). Intelligent energy-aware routing protocol in mobile IoT networks based on SDN. IEEE Trans. Green Commun. Netw..

[53] Abbas O.K., Abdullah F., Radzi N.A.M., Salman A.D., Abdulkadir S.J. (2024). Survey on Clustered Routing Protocols Adaptivity for Fire Incidents: Architecture Challenges, Data Losing, and Recommended Solutions. IEEE Access.

[54] Masood Ahmed K., Shams R., Khan F.H., Luque-Nieto M.Á. (2024). Securing Underwater Wireless Sensor Networks: A Review of Attacks and Mitigation Techniques. IEEE Access.

[55] Shi Y., Eberhart R. A modified particle swarm optimizer. Proceedings of the 1998 IEEE International Conference on Evolutionary Computation Proceedings, IEEE World Congress on Computational Intelligence (Cat. No.98TH8360).

[56] Haris M., Bhatti D.M.S., Nam H. An Improved Particle Swarm Optimization Algorithm Based on S-shaped Activation Function for Fast Convergence. Proceedings of the 2022 13th International Conference on Information and Communication Technology Convergence (ICTC).

[57] Chen H.L., Yang B., Wang S.J., Wang G., Liu D.Y., Li H.Z., Liu W.B. (2014). Towards an optimal support vector machine classifier using a parallel particle swarm optimization strategy. Appl. Math. Comput..

[58] Yao X., Liu Y., Lin G. (1999). Evolutionary programming made faster. IEEE Trans. Evol. Comput..

[59] Ghorpade S.N., Zennaro M., Chaudhari B.S., Saeed R.A., Alhumyani H., Abdel-Khalek S. (2021). Enhanced Differential Crossover and Quantum Particle Swarm Optimization for IoT Applications. IEEE Access.

[60] Hu M., Wu T., Weir J.D. (2013). An Adaptive Particle Swarm Optimization With Multiple Adaptive Methods. IEEE Trans. Evol. Comput..

[61] Lin Z. (2025). Optimizing Kernel Extreme Learning Machine based on a Enhanced Adaptive Whale Optimization Algorithm for classification task. PLoS ONE.

[62] Chen M., Wang H., Chen X. Path planning optimization of medical service robots based on PSO. Proceedings of the 2022 6th International Conference on Wireless Communications and Applications (ICWCAPP).

[63] Han W., Yang P., Ren H., Sun J. Comparison study of several kinds of inertia weights for PSO. Proceedings of the 2010 IEEE International Conference on Progress in Informatics and Computing.

[64] Liu W., Wang Z., Yuan Y., Zeng N., Hone K., Liu X. (2021). A Novel Sigmoid-Function-Based Adaptive Weighted Particle Swarm Optimizer. IEEE Trans. Cybern..

[65] Xu L., Song B., Cao M. (2021). An improved particle swarm optimization algorithm with adaptive weighted delay velocity. Syst. Sci. Control Eng..

[66] Sharmin S., Ahmedy I., Md Noor R. (2023). An energy-efficient data aggregation clustering algorithm for wireless sensor Networks using hybrid PSO. Energies.

[67] Tian P., Li K., Wu X., Yu S., Hu Y., Shi Q. (2025). Indoor Geomagnetic Matching Location Based on Iterative Local Search and Improved Particle Swarm Fusion. IEEE Robot. Autom. Lett..

[68] Rahnemay M., Farzinvash L., Zolfi M., Taherkordi A. (2024). ECMSH: An Energy-efficient and Cost-effective data harvesting protocol for Mobile Sink-based Heterogeneous WSNs using PSO-TVAC. Ad Hoc Netw..

[69] Hu Y., Zhang Y., Gong D., Sun X. (2023). Multiparticipant Federated Feature Selection Algorithm With Particle Swarm Optimization for Imbalanced Data Under Privacy Protection. IEEE Trans. Artif. Intell..

[70] Zhang L. (2022). A deployment and coverage optimization algorithm for self-powered wireless sensor networks based on hybrid swarm intelligence. IEEE Sens. J..

[71] Ou Y., Qin F., Zhou K.Q., Yin P.F., Mo L.P., Mohd Zain A. (2024). An improved grey wolf optimizer with multi-strategies coverage in wireless sensor networks. Symmetry.

[72] Song J., Hu Y., Luo Y. (2024). Wireless Sensor Network Coverage Optimization Based on the Novel Enhanced Hunter–Prey Optimization Algorithm. IEEE Sens. J..

[73] Yang S.Y., Xiang Y.H., Kang D.W., Zhou K.Q. (2024). An Improved Cuckoo Search Algorithm for Maximizing the Coverage Range of Wireless Sensor Networks. Baghdad Sci. J..

